# A multi-omics study on monozygotic twins discordant for amyotrophic lateral sclerosis and literature review underline a potential role for innate immunity and epigenetic dysregulation in disease mechanisms

**DOI:** 10.1007/s10072-026-08813-y

**Published:** 2026-02-05

**Authors:** Martina Tosi, Francesco Favero, Miriam Zuccalà, Endri Visha, Fjorilda Caushi, Nadia Barizzone, Nicola Pomella, Laura Follia, Lucia Corrado, Davide Corà, Loredana Martignetti, Maurizio Leone, Sandra D’Alfonso

**Affiliations:** 1 Department of Health Sciences, University of Piemonte Orientale UPO, Novara, Italy; 2 Center for Translational Research on Allergic and Autoimmune Diseases (CAAD), University of Piemonte Orientale, Novara, Italy; 3 Department of Translational Medicine, University of Piemonte Orientale, Novara, 28100 Italy; 4 Institut Curie, Université PSL, 26 rue d’Ulm, Paris, 75005 France; 5 INSERM, U900, 26 rue d’Ulm, Paris, 75005 France; 6 Mines ParisTech, Université PSL, 60, boulevard Saint-Michel, Paris, 75006 France; 7 Scientific Direction, Fondazione IRCCS Casa Sollievo della Sofferenza, San Giovanni Rotondo, Italy

**Keywords:** Amyotrophic lateral sclerosis, Monozygotic twins, Multi-omics approach, Data integration, Motor neuron disease, Complex disease

## Abstract

**Background:**

Amyotrophic lateral sclerosis (ALS) is a progressive neurodegenerative disorder characterized by motor neuron degeneration. Although genetic contributions to both familial and sporadic ALS (sALS) cases are well established, a substantial portion of ALS heritability remains unexplained, suggesting the involvement of other genetic and epigenetic factors.

**Methods:**

To address this gap, we have devised a comprehensive multi-omics approach in a pair of Italian monozygotic twins discordant for ALS, performing DNA methylation, transcriptomic, and whole exome sequencing (WES). We then conducted a structured literature research on ALS-discordant monozygotic twins (*n* = 45) and on case-control sALS (~ 7000 patients and ~ 3000 controls), investigated for at least one of the omics approaches.

**Results:**

Our exploratory analysis reveals distinct transcriptomic and epigenetic profiles underlying the discordant disease phenotypes in genetically identical individuals, particularly implicating immune system functions and brain development pathways. Notably, a comprehensive comparison of our results with existing literature underlined the involvement of pathways related to NK cell activation, chemokine production, and signal transduction, suggesting potential shared disease associated mechanisms across ALS cases.

**Conclusions:**

This hypothesis-generating study, although limited by the sample size, demonstrates the utility of multi-omics approaches in uncovering broader pathological insights into ALS, speculating on the possible contribution of innate immunity and epigenetic dysregulation in disease processes. This work provides a foundation for future research aimed at identifying disease-associated processes and biomarkers.

**Supplementary Information:**

The online version contains supplementary material available at 10.1007/s10072-026-08813-y.

## Introduction

Amyotrophic lateral sclerosis (ALS) is a neurodegenerative disease characterized by progressive, painless muscle atrophy due to motor neuron degeneration within the brain and spinal cord. Genetic factors are known to contribute to up to 60% of familial ALS (fALS) cases and about 15% of sporadic ALS (sALS) [1, 2] cases, with pathogenetic mutations in more than 35 genes identified as playing a role in disease pathogenesis [3–7]. Despite these advances, a substantial portion of ALS heritability remains unaccounted for, pointing to the potential involvement of additional genetic and epigenetic factors. To uncover these contributors, studies involving unique populations, such as discordant monozygotic twin pairs—who are matched for family factors (e.g., baseline genetic sequence, age, sex, and rearing environment)—have become increasingly important [8–11]. Indeed, this study design allows researchers to assess epigenetic modifications derived from environmental exposure as well as *de novo* genomic variants that may explain the discordant disease phenotype between the matched twin pair.

To date, several investigations have focused on ALS monozygotic discordant twin pairs, analyzing genome-wide DNA methylation in whole blood of monozygotic twins [12–16], blood transcriptomic profiles [13], and *de novo* genomic mutations both at the genome-wide level [14, 17] and within ALS-specific genes. These studies collectively indicate that multi-omics analysis of discordant twins represents a promising approach to gain crucial insights into underlying pathogenetic mechanisms. However, existing research has been hampered by limited cohort sizes and a narrow focus on only one or two omics dimensions at a time. Moreover, no comprehensive comparison of findings from multiple studies has been attempted to elucidate ALS associated mechanisms.

In this context, the present hypothesis-generating study considers three distinct omics data types from a novel ALS-discordant twin pair of Italian origin. To mitigate the sample size limitation, these findings have been combined for the first time with existing literature data from ALS discordant twins and sALS cases to identify possible processes correlated with the disease.

Our findings propose that specific transcriptomic and epigenetic signatures, particularly those involving innate immunity processes, as well as the Rap1 and receptor tyrosine kinase signaling pathways, potentially correlate with disease mechanisms. These differential profiles seem to point toward common disease mechanisms rather than merely reflecting unique pathogenetic trajectories in individual cases.

## Materials and methods

### Study design and sampling of monozygotic twins

We investigated a pair of Italian monozygotic twins discordant for ALS, recruited at the hospital “Casa Sollievo della Sofferenza”. One twin was diagnosed with definite ALS (El Escorial-R criteria) in 2019 at the age of 41, whereas the other is still healthy as of 2024. No comorbidities have been registered. Both twins were sampled at two different timepoints (T1 and T2), with the second blood sample taken nine months after the first one. Together with the first sampling, BMI, drinking and smoking quantity/frequency, clinical and environmental information were collected at the time of the first visit, one year after disease onset. Disease onset refers to the first symptom, weakness at the left hand.

### Sample preparation

Genomic DNA was extracted from whole blood by salting out technique; peripheral blood mononuclear cells (PBMCs) were isolated by density gradient centrifugation using Lympholyte-H (Cedarline, Burlington, NC, USA) and stored at −80 °C, while total RNA was extracted using miRNeasy Micro Kit (QIAGEN).

### Whole exome sequencing

Libraries for WES were prepared according to Agilent SureSelectQXT Kit protocol and processed on an Illumina NextSeq 550 sequencer (Illumina, San Diego, California, USA). Fastq files were aligned on the reference human genome hg19 (GRCh37) and, for each patient, a list of variations in a VCF format file (Variant Call Format) was produced by GATK software [18] and annotated by wAnnovar tool (http://wannovar.wglab.org/), using REF Seq Gene database. Moreover, we considered differences of Copy Number Variations (CNV) in the twin pair. This analysis has been performed by R package ExomeDepth [19]. See Supplementary Material 1. 

### RNA sequencing

We analyzed 8 samples as we obtained a biological duplicate from the first blood sample and a technical triplicate from the second blood sample for each twin. RNA extracted from PBMCs was used for library preparation according to Illumina TruSeq Stranded mRNA kit and processed on an Illumina NextSeq 550 sequencer (Illumina, San Diego, California, USA). STAR program [20] was used to align reads to the reference human genome GRCh38/hg38; annotations provided by the Ensembl v100 database were set as a reference for the RSEM computational pipeline [21] used for quantification of gene expression levels. Only genes with a TPM > 1 in at least one sample were considered to be expressed and underwent further analysis. DEGs were calculated using both DESeq2 [22] and edgeR [23] with |log2FC| > 1 and p.adj ≤ 0.05—the FDR was calculated using the Benjamini-Hochberg procedure—as parameters to define the statistical significance of differential gene expression. The results of the two in silico methods were combined and only overlapping DEGs were selected. Lastly, enrichment analysis was performed using Metascape [24]. Statistical and graphical computations were performed in the R environment (www-r-project.org). Validation of RNA-seq results was performed by Biorad Droplet Digital PCR (ddPCR).

### Methylation study

For each twin, we analyzed a technical replicate from each biological sample collected at T1 and T2, thus obtaining 8 samples. DNA from whole blood was converted by using bisulfite conversion technique (EZ DNA Methylation Kit) and hybridized to the Infinium Methylation EPIC Array (Illumina, San Diego, USA) that covers more than 850,000 methylation sites at the genome-wide level (reference genome hg19). After initial quality checks by Illumina GenomeStudio, data were analyzed by Chip Analysis Methylation Pipeline (ChAMP [25], package version 2.21.1), a Bio-conductor package that provides a very comprehensive analysis pipeline for EPIC array data and performs an annotation on the hg19 reference genome. Starting from IDAT files, automatic filters were applied to remove probes that failed with detection with *p*-value > 0.01 or < 3 probes in at least 5% of samples; multi-hit, non-CpG, Chromosome X/Y and SNP-overlapping probes.

In addition, possible confounders were carefully evaluated. Despite several factors listed in Table 1 that could potentially influence epigenetic mechanisms, we decided to consider only the effect of smoking on methylation status. Firstly, based on the available information, discordant twins showed major differences only in smoking status, while they were comparable for the other variables. Moreover, the exact overlap between twins’ disease status (affected vs. unaffected) and confounder status (smoker vs. non-smoker) prevented the application of standard statistical methods to adjust for confounding factors. Thus, we decided to focus only on controlling the main confounder factor and to adopt a conservative filtering strategy. Considering the effect of smoking on DNA methylation and the different smoking status of the twins, we retrieved a list [26] of 63,535 smoking-associated probes and removed them from the matrix obtained after ChAMP initial QC. Beta values were then normalized according to beta-mixture quantile normalization (BMIQ) method and corrected for batch effect (position on the array) (Supplementary Figure S7). To identify differentially methylated probes, we utilized “champ.DMP” function that implements “limma” package and allows to calculate the p-value for differential methylation analysis using a linear model on technical replicates from biological samples collected from the twins at two different time points (T1 and T2). The replicates were highly correlated (ρ > 0.99), and thus violate the assumption of sample independence required by linear modelling. Nevertheless, we chose to utilize the linear model implemented in ChaAMP package to generate exploratory results, and to cautiously consider the statistical significance represented by p-value to filter the signals for further exploratory comparisons with literature data.

Probes with |Δβ| > 0.06 and adjusted *p*-value < 0.05, calculated on Benjamini-Hochberg correction, were defined as differential methylated between two phenotypes. We also identified by “Bumphunter” [27] differentially methylated regions (DMR), defined as regions with more than seven CpG sites and false discovery rate < 0.05. Enrichment analyses on differentially methylated genes were performed by Metascape [24]. To validate epigenetic results, we performed methylation-specific droplet digital PCR combined with methylation-dependent restriction enzymes (ddMSP), according to manufacturer’s protocol. DNA methylation age was assessed using Horvath’s method (https://dnamage.clockfoundation.org/) [28]. See Supplementary Material 1.

### Structured literature search

A first structured literature search was performed in PubMed and EMBASE, with the following terms: twins, ALS, amyotrophic lateral sclerosis, and motor neuron disease. The focus was limited to studies on discordant monozygotic twin pairs, including genetic and/or transcriptomic and/or epigenetic data obtained with methods comparable to those used in our study, up to June 2024.

A second search was conducted on PubMed © including terms as: ALS, Amyotrophic Lateral Sclerosis, motor neuron disease, sporadic, PBMC, blood. Focus was limited to human studies with genetic and/or transcriptomic and/or epigenetic data obtained with methodologies comparable to the ones applied in our study, up to June 2024.

## Results

### Whole exome sequencing of an ALS-discordant twin pair

**Fig. 1:**
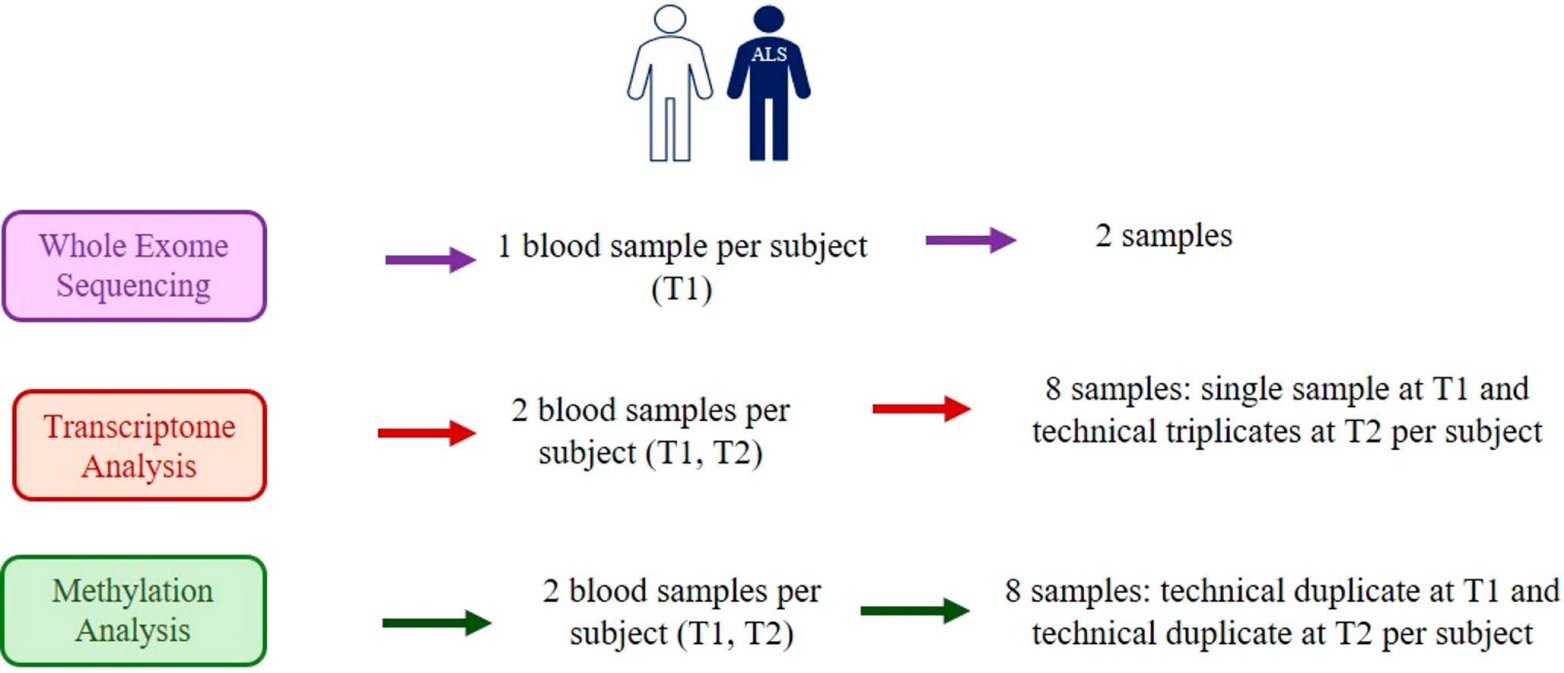
Summary of the study design. For each subject, blood samples at T1 and T2 were collected. For each omics, analyses were performed on technical or biological replicates

To identify genetic and epigenetic contributors to ALS by leveraging their genetic similarity and shared environment, we analyzed an Italian monozygotic twin pair discordant for ALS, whose main clinical features and lifestyle habits are summarized in Table 1. Blood sampling and data collection of clinical features and environmental exposures occurred at the time of the first visit to the hospital “Casa Sollievo della Sofferenza,” approximately one year after disease onset. Figure 1 graphically represents the study design.

Whole exome sequencing (WES) was performed on blood-derived DNA from both the ALS patient and the healthy co-twin. This analysis did not reveal any sequence variation classified as pathogenic or of uncertain significance (VUS) in known ALS-related genes.

To investigate post-zygotic *de novo* mutations that could be associated with ALS development, we searched for sequence variations detected exclusively in one of the two twins, focusing on both single nucleotide variants (SNVs) and copy number variations (CNVs). After quality control (QC) and frequency filtering—minor allele frequency ≤ 0.00005 in the gnomAD_EXOME_ALL database [29]— we identified 25 SNVs and 4 CNVs unique to the ALS twin, while 10 SNVs and 4 CNVs were found exclusively in the healthy co-twin (Supplementary Tables S1–S4). These variants were classified as either VUS or benign, according to the standard ACMG criteria [30], and none mapped to genes with any potential link to neurodegeneration. Thus, the involvement of these *de novo* events in ALS pathogenesis seems unlikely.

**Table 1:** Clinical features and environmental exposures of the twin pair. Data were collected at the time of the first visit, together with the first blood collection

Feature	Affected twin	Healthy twin
Current age	45	45
Sex	Male	Male
Age at diagnosis	41	-
BMI	26.99	27.68
Treatment	riluzole + tudcabil	No
Smoking status	Yes (active + passive); 3 cigars per day since 18 years of age	No (active + passive)
Drinking status	2 glasses of wine per week since 18 years of age	1glass of wine per day since 18 years of age
Work	Secretary	Bookseller
Physical activity	Judo, soccer, cycling	Judo, soccer, running
Trauma	6 non-concussive head injuries	6 non-concussive head injuries; fracture of right wrist and right arm

### Transcriptomic analysis

**Fig. 2:**
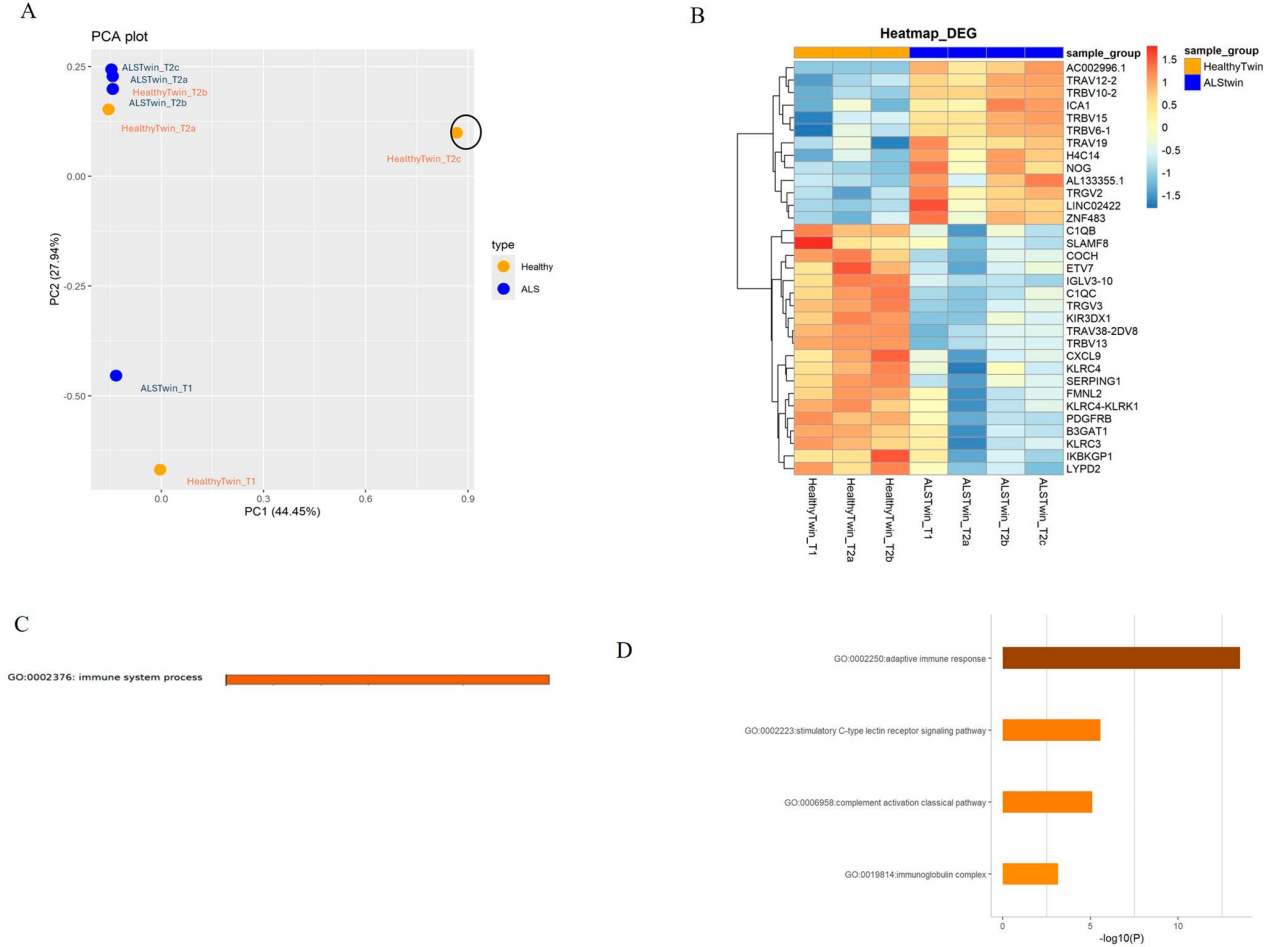
Transcriptomic analysis overview. (**A**) Principal component analysis (PCA) showing the distribution of the samples based on the time point: ALS twin samples at T1 and T2 are represented in blue, healthy twin samples at T1 and T2 are represented in orange. The outlier is circled: it corresponds to one of the technical triplicates of the healthy twin at T2 (HealthyTwin_T2c). (**B**) Heatmap showing the identified 33 differentially expressed genes (DEGs) for the healthy twin (left) and the ALS-affected co-twin (right) based on DESeq2 log2FC and p.adj. Upregulated genes are represented in the red scale, while downregulated genes are depicted in the blue scale. The gene names are displayed on the right, while clustering of genes by similar expression values is shown on the left. (**C**) Enrichment analysis on DEGs by Metascape unveiled “immune system process” as top biological process. (**D**) The bar graph shows the most associated terms identified by Metascape: GO term “adaptive immune response” is the most associated term

For each twin, RNA sequencing was performed on RNA samples collected at two different time points, T1 (single sample) and T2 (technical triplicates), nine months apart, resulting in a total of eight samples. Quality checks on the generated reads using FastQC [31] are shown in Supplementary Figure S1. Principal component analysis (PCA) shows the distribution of the samples based on the time point: for this reason, “HealthyTwin_T2c” was identified as an outlier and removed (Fig. 2A). Differential expression analysis was carried out on the remaining seven samples—four samples from the ALS twin and three from the healthy co-twin. By using DESeq2 [22] and edgeR [23] and filtering for adjusted p-value ≤ 0.05 and |log2FC| > 1, we identified 39 and 45 differentially expressed genes (DEG) respectively. We then combined the results obtained by the two methods, selecting 33 overlapping genes that were considered for functional analyses. Among these 33 DEGs, 20 were upregulated and 13 downregulated in the ALS twin compared to the healthy co-twin (Fig. 2B and Supplementary Figure S2). The list of DEGs is provided in Supplementary Material 2.

Enrichment analysis by Metascape [24] (Fig. 2C) revealed that the “immune system process” was the most enriched biological pathway. More specifically, enriched terms included “adaptive immune response” (Fig. 2D), probably as a consequence of somatic rearrangement of immune genes belonging to the *TRAV*, *TRBV*, and *TRGV* gene families, which map to the variable (V) region of the T cell receptor (TCR) alpha (α), beta (β), or gamma (γ) chain, respectively. Such rearrangements are expected to differ between individuals, even between monozygotic twins, whereas innate immune-related terms would be less affected. Interestingly, among the DEGs, we observed genes involved in innate immune response, encoding early complement components (*C1QB* and *C1QC*), regulators of the complement cascade (*SERPING1*), chemokines (*CXCL9*), and proteins involved in NK cell activation, such as the self-ligand receptor of the signaling lymphocytic activation molecule (*SLAMF8*), and members of the killer cell lectin-like receptor subfamily C (*KLRC4*,* KLRK1*, and *KLRC3*).

To validate the RNA-seq results with a different semi-quantitative method, we performed digital droplet PCR (ddPCR) on three upregulated genes (*PF4V1*,* JUN*,* PKIB*), identified by DESeq2, and three downregulated ones (*KLRC3*,* SERPING1*,* C1QB*), identified by both DESeq2 and edgeR, in the ALS twin compared to the healthy co-twin, based on TPM counts. *HPRT1* was chosen as the housekeeping gene. We confirmed the expression trends of these genes (Supplementary Figure S3): in the ALS twin (vs. the healthy co-twin), the measured concentrations (expressed as copies/µl) were 32.7 vs. 25.5 for *PF4V1*, 45 vs. 33 for *JUN*, and 3.53 vs. 1.38 for *PKIB*. On the other hand, *KLRC3*,* SERPING1*, and *C1QB* were expressed at higher levels in the healthy twin compared to the ALS one (49.3 vs. 33 copies/µl for *KLRC3*, 26 vs. 12.4 for *SERPING1*, and 18.8 vs. 6.93 for *C1QB*).

### Methylation study analysis

We next performed a genome-wide methylation analysis on eight samples consisting of technical replicates from biological samples collected from the twins at two different time points (T1 and T2), applying a linear regression analysis implemented in ChAMP [25] package, despite the violation of independence assumptions, with the aim to perform an exploratory analysis.

**Fig. 3:**
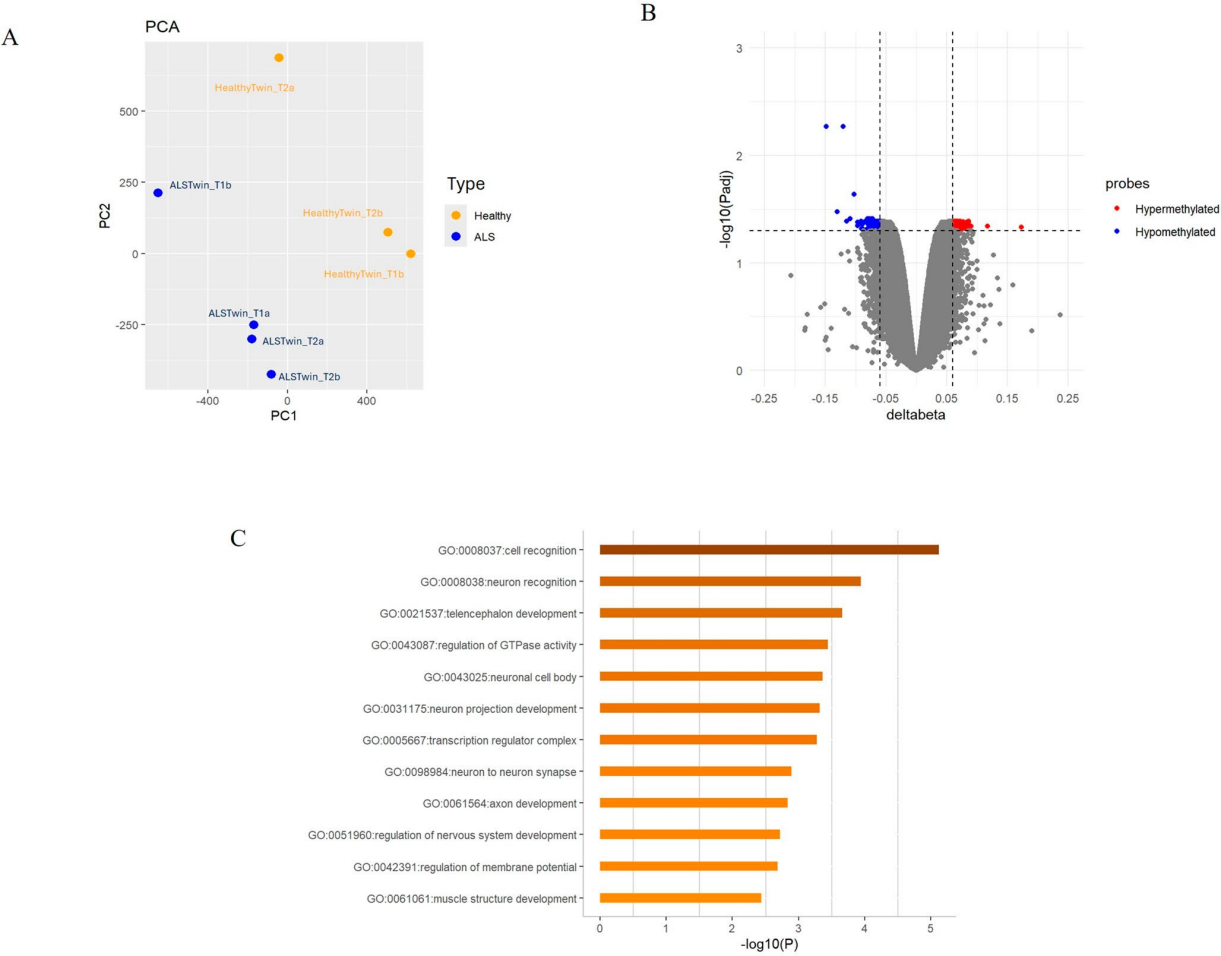
Methylation analysis overview. (**A**) The PCA shows the distribution of the samples, labelled by their disease status (in blue ALS twin, in orange healthy twin) and timepoint (T1, T2), after outlier (HealthyTwin_T1a) and smoking-associated probes removals. (**B**) Volcano plot showing the probes distribution according to Δβ on x-axis and -log10(adjusted p-value) on y-axis before smoking-associated probes removal. Probes with Δβ > 0.06 are considered hypermethylated (red), while those with Δβ < −0.06 are hypomethylated (blue). (**C**) After removing the outlier and the smoking-associated probes, the analysis identified 89 differentially methylated probes (DMPs) mapping to 64 genes. The bar graph highlights the most significantly associated terms identified through enrichment analysis by Metascape of these 64 genes: the most enriched terms are brain-related and involve cellular and developmental processes

QC checks using ChAMP are shown in Supplementary Figure S4. PCA identified an outlier corresponding to sample 1 (HealthyTwin_T1a), while the remaining samples clustered into two subgroups based on their position on the array (Supplementary Figure S5). After removing the outlier, the analysis was conducted on the remaining seven samples— four samples from the ALS twin and three from the healthy co-twin. After QC, we obtained 741,329 probes; data were then normalized and corrected for batch effect. By applying filters of adjusted *p*-value ≤ 0.05 and | Δβ | > 0.06, we identified 250 differentially methylated probes (DMPs) mapping to 190 genes: 108 DMPs were hypermethylated (positive Δβ), whereas 142 were hypomethylated (negative Δβ) in the ALS twin compared to the healthy co-twin (Fig. 3B). The list of DMPs is provided in Supplementary Material 3.

To technically validate the top DMP using an independent method, we analyzed two probes through methylation-specific ddPCR: cg18454685 in *CACNA1G* and cg27533288 in *VAX1.* These probes were selected because, among the DMPs, the two cytosines of interest were located within the restriction sites of specific methylation-sensitive restriction enzymes (BstUI and MwoI). The results confirmed hypomethylation in the ALS twin, as shown in Supplementary Figure S6.

*CACNA1G* and *VAX1* are genes known to be differentially methylated in relation to smoking exposure. More generally, considering the effect as confounder factor of smoking on DNA methylation and the twins’ different smoking status, from the list of probes that passed QC, obtained from the eight samples, we removed a list of 63,535 smoking-associated probes (Supplementary Material 3). These were derived from the largest comprehensive study evaluating smoking exposure interactions on DNA methylation, performed using the same EPIC array utilized in our study [26]. As previously observed, PCAs pointed out that samples grouped according to their position on the array and, after normalization and batch correction, HealthyTwin_1a was confirmed as outlier (Supplementary Figure S7 A-B-C-D). Firstly, we removed the outlier conducting the analyses on a total of seven samples; secondly, from the initial 741,329 probes, we removed the 63,535 smoking-associated ones (Supplementary Material 3) and performed the entire ChAMP pipeline, including normalization and batch correction (Fig. 3A, Supplementary Figure S7 E-F-G). By applying the same filters of adjusted p-value ≤ 0.05 and | Δβ | > 0.06, we identified 89 DMPs mapping to 64 genes: 28 DMPs were hypermethylated (positive Δβ), while 61 were hypomethylated (negative Δβ) in the ALS twin compared to the healthy co-twin. The list of DMPs is provided in Supplementary Material 3. In addition, six DMRs related to three genes were identified (Supplementary Material 4). None of these genes belong to ALS and neurodegeneration-related KEGG pathways [32].

We then performed an enrichment analysis by Metascape [24] on the 64 genes which revealed an involvement of terms related to cellular and developmental processes, such as GO:0008037 “cell recognition”, GO:0043087 “regulation of GTPase activity”, GO:0021537 “telencephalon development”, and GO:0061061 “muscle structure development”. Furthermore, brain-related terms were also enriched, including GO:0008038 “neuron recognition”, GO:0051960 “regulation of nervous system development”, GO:0061564 “axon development”, and GO:0043025 “neuronal cell body” (Fig. 3C). Moreover, we separately selected all the genes in which hyper- and hypomethylated DMPs mapped and performed two individual enrichment analyses using Metascape [24] to identify possible pathways linked to hyper- and hypomethylation (Supplementary Figure S8). Analysis of hypomethylated genes in ALS twin highlighted terms linked to distinct cellular and regulatory processes: GO:0008037 “cell recognition”, GO:0005667 “transcription regulator complex”, and GO:0043087 “regulation of GTPase activity”. The few hypermethylated genes were enriched in GO:0050767 “regulation of neurogenesis”.

Lastly, we calculated the epigenetic age using the algorithm developed by Steven Horvath [28]. The calculated DNA methylation (DNAm) age was similar for both twins, averaging 44 years, which was close to their actual age at sampling (41 years).

### Multi omics data analysis

After analyzing each omics dataset individually, we combined the results searching for common terms. Specifically, we examined shared genes across methylation, transcriptomic, and genetic top signal data. However, no common genes were identified, as shown in the Venn diagram (Supplementary Figure S9).

### Structured literature search and comparative analysis

To further optimize the identification of ALS-related signatures through the analysis of discordant monozygotic twins, we combined and compared our findings with published data by performing a structured literature research on PubMed Central and EMBASE. Beyond providing a general overview of the results achieved so far, this approach aimed to mitigate the sample size limitation, both of our study and of literature ones. In addition, the comparison with literature data was intended to provide robust support for the results obtained from our study. We identified six studies [12–17] including a total of 45 ALS discordant twin pairs or triplets (Supplementary Table S5), each investigating at least one of the omics approaches described in our study, and compared the results to those identified in the Italian twin pair. Since raw data were not available for all studies, we relied on lists or tables of DMPs, genes, and pathways published in “Results” sections or supplementary materials of each paper. For each omics layer, we specifically searched for genes or biological processes prioritized in the literature and compared them with those identified in our Italian twin pair. Upon reviewing the genetic results from three studies [14, 16, 17], no overlapping variants were found with those previously reported from our study (Supplementary Tables S1 and 2). In good agreement, Steinberg et al. [17] reported no pathogenic discordant SNVs or CNVs in coding or regulatory regions among ALS twin siblings.

**Fig. 4:**
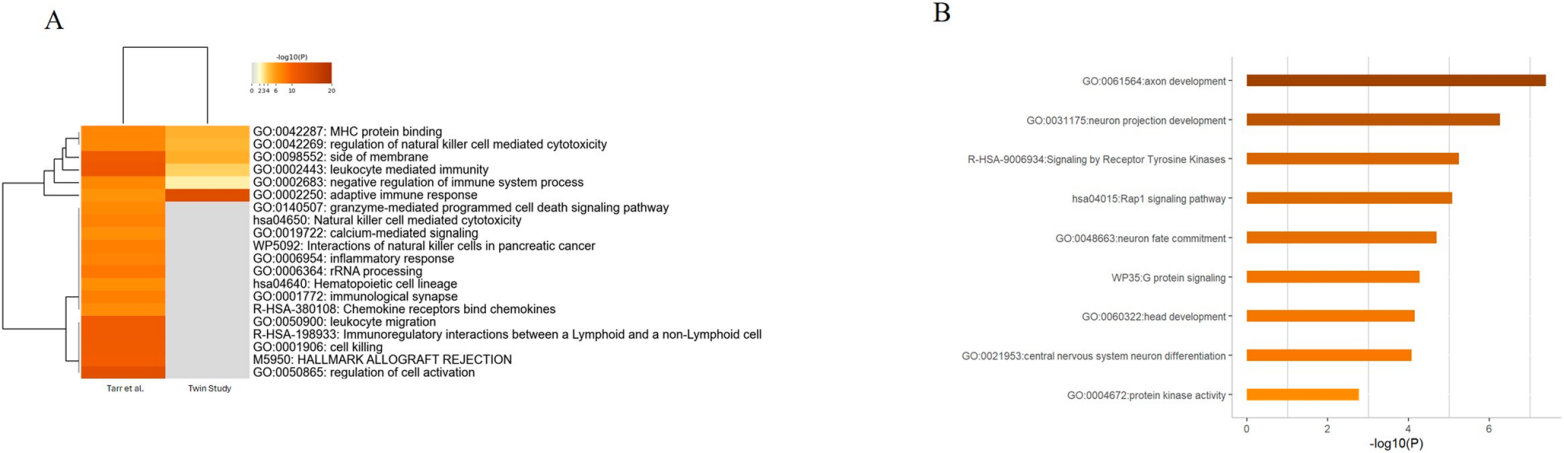
Comparative enrichment analysis based on literature comparisons. (**A**) Heatmap showing the results of the enrichment analysis by Metascape comparing 750 DEGs identified in the study by Tarr et al. [13] (left column) and 33 DEGs identified in our study (right column). The most enriched terms are linked to immune system processes, such as “adaptive immune response” and “leukocyte-mediated immunity”. (**B**) The bar graph shows the enrichment analysis by Metascape of the 69 differentially methylated genes shared across all literature twin studies and our twin study. Brain-related and signaling processes resulted as the most significantly enriched, including as representative GO:0061564 “axon development”, GO:0021953 “central nervous system neuron differentiation”, hsa04015 “Rap1 signaling pathway” and R-HSA-9,006,934 “signaling by receptor tyrosine kinases”

The only transcriptomics-focused study by Tarr et al. [13] reported 750 DEGs in male ALS twins, with only four overlapping the 33 DEGs identified within our twin pair: *B3GAT1*,* LYPD2*,* PDGFRB*, and *KLRC3*. Enrichment analysis on the 750 DEGs by Metascape confirmed the involvement of immune system processes (e.g., leukocyte-mediated immunity, leukocyte migration, regulation of natural killer cell mediated cytotoxicity, adaptive immune response, and MHC protein binding), consistent with the results from our Italian twin pair (Fig. 4A). In particular, the DEGs linked to the “regulation of natural killer cell-mediated cytotoxicity” term from the literature [13] included genes encoding proteins involved in similar biological function as those identified in our study, such as chemokines (*CCL3*,* CCL4*, and *CCL5*), proteins promoting the release of pro-inflammatory chemokines (*CSF1*, and *IL34*), and proteins mediating NK cell activation (*NKG7* and *CD160*). Among these, *KLRC3* and *PDGFRB* were shared between both studies.

Epigenetic data at genome-wide level has been reported for the majority of the identified literature papers [12–16], generated mainly by Illumina 450–850 K Infinium methylation arrays and by reduced representation bisulfite sequencing (RRBS). For each paper, we compared the list of DMPs or genes prioritized in our study with those reported in the literature to identify shared signals or pathways. For instance, the enrichment analyses on RRBS outliers by Young et al. [15] and Tarr et al. [13] revealed enrichment in various biological functions linked to neurobiological and developmental pathways, including GO:0000902 “cell morphogenesis”, GO:0051960 “regulation of nervous system development”, GO:0061061 “muscle structure development”, GO:0007420 “brain development”, and GO:0003700 “DNA binding transcription factor activity”. These terms largely matched our previously described ontology findings (Fig. 3C).

Altogether, we identified 16 differentially methylated genes shared between the literature studies and our results (Supplementary Material 3). To extract common pathways associated with the disease, we applied an inclusive strategy by filtering our results for adjusted *p*-value ≤ 0.1 and | Δβ | > 0.06; then, we selected only the differentially methylated genes in common with those reported in the literature [12–16], identifying a subset of 69 genes (Supplementary Material 3). Enrichment analysis by Metascape on this common gene set (Fig. 4B) uncovered GO terms primarily linked to developmental processes (GO:0032502), particularly those related to brain- development. Among the most significantly associated GO biological processes based on *p*-value, we observed GO:0061564 “axon development”, GO:0021953 “central nervous system neuron differentiation”, and GO:0031175 “neuron projection development” (Fig. 4B). Interestingly, pathways such as hsa04015 “Rap1 signaling pathway” and R-HSA-9006934 “signaling by receptor tyrosine kinases,” which are associated with biological processes contributing to disease mechanisms, were also highlighted [33, 34].

As a final comparison, we looked for common differentially methylated genes shared across all studies [12–16]. However, no common genes were identified.

In addition to methylation status, DNA methylation (DNAm) age was reported in each study. Interestingly, aside from our results and those published by Tazelaar et al. [14], other studies have shown that ALS-affected twins tend to show an older epigenetic age compared to their unaffected counterparts.

To strengthen the results obtained from the aforementioned comparisons of discordant twins and to confirm potential disease mechanisms, we retrieved several studies [35–40] on sALS subjects and controls who underwent genetic and/or transcriptomic and/or epigenetic investigations including a total of 7,046 sALS and 3,110 controls (Supplementary Table S6).

Regarding transcriptomic results, we extracted 87 DEGs [35], none of which overlapped with the 33 DEGs from our twin study. Only two genes, *PDCD1* and *GOLGA2P5*, were shared with the 750 DEGs from Tarr et al. [13]. Enrichment analysis revealed GO terms involved in the regulation of several processes, including glucose import, cell morphogenesis, proteolysis, sequence-specific DNA binding, transport of small molecules, and cancer development. In a second study on a larger cohort by Grima et al. [37], 245 DEGs were identified: enrichment analysis conducted by Metascape, Kyoto Encyclopedia of Genes and Genomes (KEGG) [32], and QIAGEN Ingenuity Pathway Analysis (IPA, QIAGEN Inc., https://digitalinsights.qiagen.com/IPA) revealed involvement of metabolic pathways, cell stress, and immune response (i.e., GO:1902107 “positive regulation of leukocyte differentiation”, GO:0050900 “leukocyte migration”, and GO:0070098 “chemokine-mediated signaling pathway”). Similar immune-related processes (i.e., “regulation of leukocyte migration”, “regulation of leukocyte chemotaxis”, and “different interleukin signaling pathways”) were also identified in the transcriptomic ALS signature from PBMCs described in a recent German case-control study [40]. Immune response was also positively enriched in ALS patients in the study by Liguori et al. [38], which reported 1,566 upregulated genes. Altogether, the role of immune response was partially reconfirmed by the literature studies: the three largest ones [37, 38, 40], accounting for over 170 sALS and 80 controls, consistently revealed enrichment of genes involved in immune response. Conversely, the two smaller studies, accounting for a total of 28 sALS patients and 15 controls, failed to highlight any involvement of immune responses [35, 39], possibly due to limited statistical power resulting from small sample sizes.

**Fig. 5:**
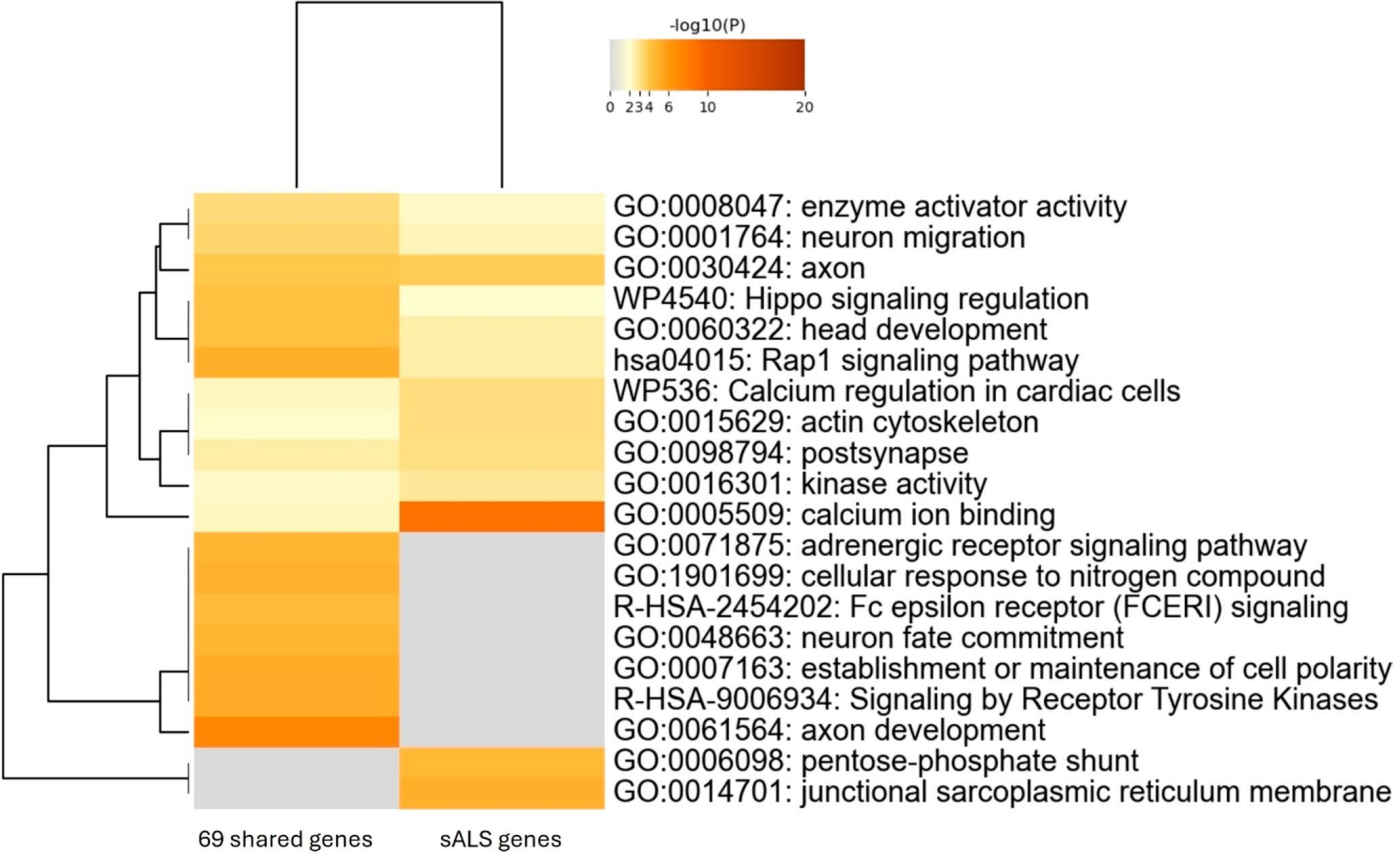
Comparative methylation analysis. The heatmap represents the result of the enrichment analysis comparing 69 differentially methylated genes across twin studies and 138 differentially methylated genes retrieved by Feró et al. study on sporadic ALS (sALS). The most enriched terms are linked to calcium ion binding (GO:0005509), brain component (“axon” GO:0030424) and signaling pathway (“Rap1 signaling pathway” hsa04015)

For genetic data, after applying the same filters as in our analysis, no common variants were identified between our study and those found in the clinical exome sequencing performed by Feró et al. [36], while 138 differentially methylated genes were retrieved from the same study [36] after filtering for |Δβ| > 0.1 and *q*-value < 0.05. A comparative pathway analysis between the 69 differentially methylated genes shared across twin studies and 138 sALS genes confirmed the enrichment of brain component-related terms (i.e., GO:0030424 “axon” and GO:0098794 “post synapse”) and highlighted the “Rap1 signaling pathway” (hsa04015). In addition, it revealed the involvement of processes linked to calcium channel activity (Fig. 5).

Terms related to signal transduction, tyrosine kinase activity, and neural-muscular structure or function (i.e., WP4223 “Ras signaling”, GO:0005884 “actin filament”, and GO:0004714 “transmembrane receptor protein tyrosine kinase activity”) also emerged from a comparison of differentially methylated genes between sALS and controls in a study aimed to characterize methylation profiles associated with clinical heterogeneity in disease progression and survival among sALS patients [41]. Furthermore, the 39 differentially methylated genes specifically associated with progression rate and survival time in sALS patients were enriched in GO terms linked to brain component, specifically axonal structure and synapses, as observed in our study and in other sALS datasets [36, 40].

Another epigenome-wide study (EWAS) on 32 sALS and healthy controls (HCs) identified 34 DMPs mapping in 13 genes and 12 DMRs [42]. However, none of these overlapped with the data from our twin pair or with the common genes identified from twins’ literature studies. Lastly, we considered one of the largest EWAS published to date [43], conducted on 6,763 ALS patients and 2,943 controls from four different cohorts (MinE 450 K and EPIC, AUS 1, and 2). After applying a genome-wide threshold (*p* < 9 × 10^− 8^), 44 DMPs annotated to 42 genes were associated with ALS. Gene set enrichment analysis, based on both nearest genes and cis-eQTMs annotated to each tested position, revealed cholesterol/steroid biosynthesis–related pathways and immune-related pathways (“cytokine–cytokine receptor interaction” and “natural killer cell-mediated cytotoxicity”) as the most enriched terms. However, none of the ten most associated sites and genes driving the enrichments overlapped with the 69 differentially methylated genes identified from twin studies.

## Discussion

In this exploratory study, we used monozygotic twin pair discordant for ALS as a model system to identify potential disease risk factors and to elucidate possible post zygotic somatic events contributing to sporadic ALS susceptibility. To this end, we performed WES, transcriptomic, and genome-wide methylation studies, analyzing each omics dataset individually and, for the first time, we attempted to consider the three datasets in combination. The twin pair tested negative for pathogenetic sequence variants in known ALS genes, and there were no other family members reported to have ALS.

In good agreement with previous studies [17], WES analysis revealed that none of the SNVs and CNVs detected in only one of the twin pair were predicted to be pathogenic or likely pathogenic. Furthermore, no pathogenic variants were found in genes identified from whole genome sequencing (WGS) in 21 twin pairs reported in the literature [14] or from clinical exome sequencing of seven sporadic ALS patients [36]. These findings suggest that, based on genetic sequencing data, no variants of definitive pathological significance were identified that could explain a *de novo* somatic mutation causing ALS in only one of the twins. Conversely, the comparison of transcriptomic and epigenetic data between the Italian twin pair and those from the literature published data revealed several shared biological processes and pathways with potential relevance to disease development. Indeed, enrichment analysis combining the 33 DEGs identified in our study with the 750 DEGs reported by Tarr et al. [13] pointed to immune system-related processes, specifically GO:0002250 “adaptive immune response” and GO:0002443 “leukocyte mediated immunity”. Similarly, changes in both innate and adaptive immune cell populations have been linked to disease progression in mouse models and ALS patients, likely as a result of neuroinflammatory processes triggered by the progressive degeneration of motor neurons and axons in the spinal cord [44, 45].

The DEGs associated with the “regulation of natural killer cell mediated cytotoxicity” GO term from our enrichment analysis encode proteins involved in innate immunity, which is known to be implicated in neurodegeneration in ALS [45, 46].

Among these, proteins involved in NK cell activation appear to be implicated in our analysis. Several studies have demonstrated the role of NK cells infiltrating the CNS in regulating neuroinflammatory processes in neurodegenerative diseases [47–49], suggesting that motor neuron degeneration may stem from direct interactions with NK cells, which release perforin and granzyme B, thereby exerting neurotoxic effects on motor neurons [50].

Interestingly, the immune system dysregulation between the discordant twins observed from gene expression analyses in this study was partially supported by transcriptomic analyses conducted on PBMCs from sALS patients. Indeed, the three largest literature studies [37, 38, 40], accounting for over 170 sALS patients and 80 HCs, revealed an enrichment of genes involved in immune response. These immune signatures may reflect secondary effects, as environmental factors, and may have contributed to disease development.

From an epigenetic perspective, enrichment analysis of the 69 differentially methylated genes shared between the literature studies and our results revealed GO terms strongly associated with developmental processes (GO:0032502), particularly brain-related ones, such as GO:0061564 “axon development” and GO:0021953 “central nervous system neuron differentiation”. These findings were further supported not only by comparisons with other available studies on ALS-discordant monozygotic twins or triplets, but also by a study conducted on sALS patients focused on associations with disease progression and survival [41]. This is particularly significant, as the epigenetic analysis conducted on DNA extracted from whole blood indicated that the most enriched terms were brain-related processes, highlighting the involvement of the primary tissue affected by the disease. These data are consistent with a study by Kühlwein et al. [40], which for the first time linked epigenetic marks of a neurodegenerative disease with physiological relevance to the affected tissues in the CNS and the periphery. In particular, by analyzing chromatin accessibility of PBMCs and motor cortex tissues from sALS patients, they identified an ALS-associated epigenetic signature (i.e., “epiChromALS”) enriched in CNS neuronal pathways and detectable also in peripheral blood cells.

In addition, processes most strongly associated in twin studies included the “Rap1 signaling pathway” (hsa04015) and “signaling by receptor tyrosine kinases” (R-HSA-9006934) terms. At brain level, Rap1 plays a role in neuron migration and maturation, as well as in the plasticity of dendritic spines and synapses. Rap1 GTPases function upstream of the MAPK pathway activating the Raf/MEK/ERK cascade [34] that, when impaired, promotes neurodegenerative processes by altering neuronal survival, synaptic function, and cellular stress responses. Moreover, the Rap1/B-Raf/ERK pathway is induced by Ca^2+^ signal upstream of Rap1 activation, underscoring an important crosstalk between Rap1 and Ca^2+^ signaling pathways. This interplay controls critical cellular functions such as synaptic plasticity, gene expression, and neuronal survival [51].

Given that Ca^2+^-dependent events are central to the pathogenesis of several neurodegenerative disorders, Rap1 may represent an important therapeutic target for the treatment of neurodegenerative disorders associated with calcium signaling aberrations. Interestingly, both the “Rap1 signaling pathway” and calcium-related terms such as GO:0005509 “calcium ion binding” emerged from the comparison between 69 differentially methylated genes across twin studies and the 138 differentially methylated genes identified in sALS, suggesting a possible contribution of these pathways to the general disease mechanism.

Another recurrent enriched pathway, observed in both the epigenetic analyses on discordant twins and on sALS and matched controls [41], involves receptor tyrosine kinase (RTK) activity and signaling. The role of specific tyrosine kinases encoded by potential ALS-related genes, and of other kinases involved in pathways linked to selective motor neuron death, is well established [33]. One of the cascades modulated by RTKs includes the MAPK pathway, which also involves the aforementioned Rap1 GTPase. Therefore, the Rap1 and RTK pathways, identified across discordant twins and sALS studies, could be partially interconnected, and their involvement in general ALS mechanisms may be speculated.

Of note, the differential methylation profile in genes related to brain pathways was detectable in blood cell analysis, suggesting that, in the context of a neurodegenerative disorder, blood epigenetic changes might reflect processes occurring also in the affected tissue or organ.

We further investigated the relationship between the twin’s chronological and biological ages, but no discrepancies were observed. These results slightly contrast with previous reports, where asymptomatic co-twins tended to exhibit a younger epigenetic age compared to their ALS-affected siblings [12–16].

To better understand the biological bases of a multifactorial disease such as ALS, we explored multi-omics contributions by searching for shared genes across the genetic, transcriptomic, and epigenetic datasets. However, this analysis did not reveal any common genes among the three layers.

Despite the promising findings, the major limitation of our study remains that the analysis has been conducted on a single pair of discordant twins for ALS, which restricted our ability to meaningfully perform statistical analyses and integrate data in particular with “early” integration approaches. In addition, the study design did not allow a proper data adjustment for confounding factors. Specifically, the Italian twin pair mainly differed in smoking status, and the influence of smoking on DNA methylation and its associated epigenetic signature is well recognized [26]. Due to the limited sample size, we could not apply standard statistical corrections; nevertheless, we calculated p-values that must be interpreted with caution in the context of an exploratory investigation. However, we attempted to mitigate this issue by removing probes associated with a validated smoking-related signature [26].

It is important also to note that, when applying the ChAMP package for methylation analysis, we performed a linear regression analysis without properly accounting for sample independence, despite the high correlation among technical replicates. Considering the extremely limited sample size, this choice was justified by the need to preliminarily identify exploratory methylation differences, acknowledging that the results might overestimate statistical significance due to the violation of the independence assumption. However, this limitation has been partially overcome by comparison with literature studies that provided support for our results.

Lastly, while the use of blood rather than CNS tissue was practical and non-invasive, it implies that findings may not fully capture disease-specific changes occurring in the nervous system.

## Conclusions

In conclusion, this exploratory study aimed to contribute in elucidating the molecular mechanisms underlying ALS using the discordant monozygotic twin model. Analyzing one single pair of discordant for ALS monozygotic twins affected the statistical robustness and significance of the obtained results, preventing the application of data adjustment, proper data integration and statistical corrections. To attenuate the limitation of the small sample size and to provide contextual validation for the findings obtained from the analysis of one twin pair, for the first time we combined our results from the Italian twin pair with data from six studies conducted on 45 twin pairs or triplets, and from nine studies on 7,046 sALS and 3,110 controls. Our results should be considered speculative and suggest differential transcriptomic and epigenetic profiles observed in discordant twins that potentially correlate with ALS, rather than merely reflect individual disease processes.

To the best of our knowledge, this study is the first to analyze ALS-discordant monozygotic twins across three omics layers. Despite the pilot nature of this study and its inherent sample size limitations, the comprehensive molecular analysis conducted aimed to offer a novel perspective on how multi-omics data can enhance our understanding of complex neurodegenerative disorders.

## Supplementary Information

Below is the link to the electronic supplementary material.

Supplementary Material 1 (PDF 1.17 MB)

Supplementary Material 2 (XLSX 20.5 KB)

Supplementary Material 3 (XLSX 6.34 MB)

Supplementary Material 4 (XLSX 12.3 KB)

Supplementary Material 5 (R 1.65 KB)

Supplementary Material 6 (PL 928 bytes)

Supplementary Material 7 (PL 69 bytes)

Supplementary Material 8 (R 3.02 KB)

Supplementary Material 9 (R 458 bytes)

Supplementary Material 10 (PL 360 bytes)

Supplementary Material 11 (TXT 1.02 MB)

Supplementary Material 12 (TXT 129 bytes)

Supplementary Material 13 (TXT 658 bytes)

## Data Availability

The datasets generated during the current study are available in the European Genome-phenome Archive (EGA) repository, which is hosted by the EBI and the CRG, under accession No. EGAD00010002716, EGAD50000001329, and EGAD50000001330. Further information about EGA can be found at https://ega-archive.org and “The European Genome-phenome Archive of human data consented for biomedical research”.
