# Supplementary material for: A multi-omics study on monozygotic twins discordant for amyotrophic lateral sclerosis and literature review underline a potential role for innate immunity and epigenetic dysregulation in disease mechanisms
Source: Neurol Sci. 2026 Feb 5;47(3):230. doi: 10.1007/s10072-026-08813-y (PMC12872720; doi:10.1007/s10072-026-08813-y)
Supplement: Supplementary file 1 — Supplementary Material 1 (PDF 1.17 MB) [file 10072_2026_8813_MOESM1_ESM.pdf]

A multi-omics study on monozygotic twins discordant for amyotrophic lateral sclerosis and literature review underline a potential role for innate immunity and epigenetic dysregulation in disease mechanisms, *Neurological Sciences*, Martina Tosi, Francesco Favero, Miriam Zuccalà, Endri Visha, Fjorilda Caushi, Nadia Barizzone, Nicola Pomella, Laura Follia, Lucia Corrado, Davide Corà, Loredana Martignetti, Maurizio Leone, Sandra D'Alfonso*

Corresponding author:

Sandra D'Alfonso

Department of Health Sciences,

Via Solaroli 17, 28100, Novara, Italy

University of Piemonte Orientale UPO,

Novara, Italy

sandra.dalfonso@med.uniupo.it

Supplementary Materials

Whole Exome Sequencing: SNV

We performed Whole Exome Sequencing (WES) on the DNA extracted from whole blood of the healthy and the ALS twin. The analysis was restricted to single nucleotide variants (SNVs) passing the quality control filter (PASS), with an allele frequency on GnomAD_exome_all ≤ 0. 00005% or without frequency. These variants were studied for their position in the gene, their function, the prediction of their pathogenicity assessed by Franklin (https://franklin.genoox.com) and Varsome[1] according to the ACMG guidelines. In the ALS twin, we identified 162 variants, of which only 25 passed quality controls. Among those 25 variants, 15 mapped in an exonic position with different functions: 8 nonsynonymous, 5 synonymous, 1 nonframeshift deletion and 1 nonframeshift insertion. For what concerned the prediction of the effect Aaccording to Varsome or Franklin, 18 variants were classified as variant of unknow significance (VUS) and 7 as likely benign (Supplementary Table S1). In the healthy twin, 109 different variants were identified, but only 10 passed the quality filters. Of these variants, 8 mapped in an exonic position: 4 nonsynonymous, 3 synonymous, 1 nonframeshift deletion (Supplementary Table S2). According to Varsome or Franklin, 7 variants were classified as VUS and three as benign.

Chromosome Coordinates (hg19)	Ref	Alt	Gene	Function	Prediction
chr2_27604528	A	G	*PPM1G*	nonsynonymous SNV	VUS

chr2_95943174	G	A	*PROM2*	nonsynonymous SNV	LB
chr2_96517885	G	A	*ANKRD36C*	synonymous SNV	LB
chr2_96521438	A	G	*ANKRD36C*	nonsynonymous SNV	VUS
chr2_98128172	C	A	*ANKRD36B*	nonsynonymous SNV	VUS
chr2_98128258	G	C	*ANKRD36B*	synonymous SNV	LB
chr2_98128313	G	A	*ANKRD36B*	nonsynonymous SNV	VUS
chr2_98128364	T	C	*ANKRD36B*	nonsynonymous SNV	VUS
chr2_99938522	T	C	*TXNDC9*	synonymous SNV	LB
chr2_120125173	C	T	*DBI*	nonsynonymous SNV	VUS
chr2_227953507	C	T	*COL4A4*	synonymous SNV	LB
chr3_195510865	AGAGGGGTGGTG TCACCTGTGGATG CTGAGGGAGTGTC GGTGACAGGT	-	*MUC4*	nonframeshift deletion	VUS
chr9_69423792	A	T	*ANKRD20A4*	nonsynonymous SNV	VUS
chr15_23686113	-	TGCTCTT GCATCTTCTCG	*GOLGA6L2*	nonframeshift insertion	VUS
chr19_1881473	C	T	*ABHD17A*	synonymous SNV	LB
chr2_9568997	T	C	*CPSF3*	intronic	VUS
chr2_11364442	C	T	*ROCK2*	intronic	VUS
chr2_20507725	G	A	*PUM2*	intronic	VUS
chr2_45801874	G	A	*SRBD1*	intronic	VUS
chr2_109278689	G	A	*LIMS1*	intronic	LB
chr2_153004660	T	C	*STAM2*	intronic	VUS
chr2_201721592	A	T	*CLK1*	intronic	VUS
chr2_214161948	G	T	*SPAG16*	intronic	VUS
chr5_87516543	AG	-	*TMEM161B*	intronic	VUS
chr19_55250906	C	G	*KIR2DL3*	intronic	VUS

Supplementary Table S1. Description of the 25 ALS twin variants that passed quality control filters. Information about position, reference and alternative allele, gene, function and Varsome or Franklin prediction are reported. VUS= Variant of uncertain significance; LB= likely benign

Chromosome Coordinates (hg19)	Ref	Alt	Gene	Function	Prediction
chr1_117158934	C	T	*IGSF3*	synonymous SNV	LB
chr10_126683162	C	T	*CTBP2*	nonsynonymous SNV	VUS
chr7_151038821	A	T	*NUB1*	upstream	VUS
chr9_96438998	T	C	*PHF2*	synonymous SNV	VUS
chr15_23686089	CTC	-	*GOLGA6L2*	nonframeshift deletion	VUS
chr15_43910193	C	T	*STRC*	synonymous SNV	VUS
chr16_70896033	C	T	*HYDIN*	nonsynonymous SNV	VUS
chr19_17439719	C	A	*ANO8*	nonsynonymous SNV	LB
chr19_18109187	C	T	*KCNN1*	nonsynonymous SNV	VUS
chr20_29624127	T	G	*FRG1BP*	intronic	LB

Supplementary Table S2. Description of the 10 healthy twin variants that passed quality control filters. Information

about position, reference and alternative allele, gene, function and Varsome or Franklin prediction are reported. VUS= Variant of uncertain significance; LB= likely benign

Whole Exome Sequencing: CNV

We searched for twin-specific variants also among the Copy Number Variations (CNV), reporting only CNVs that showed differences in the start, end or both, over a certain arbitrary threshold (set at 60%). Then, CNVs typical for each twin were classified as benign, pathogenic or of uncertain significance by ClassifyCNV[2] Scores. The CNVs detected only in the ALS twin comprised 3 deletions and 1 duplication of uncertain significance and 3 duplications predicted as benign (Supplementary Table S3). Regarding the CNV detected only in the healthy co-twin, we identified 2 deletions and 2 duplications of uncertain significance, together with 1 duplication and 1 deletion predicted as benign (Supplementary Table S4).

Chromosome Coordinates (hg19)	Size	Type	Prediction	Gene	Position
chr1:12595-15930	3335	deletion	Uncertain significance	*DDX11L1*	Exon-Intron
chr1:69063-129201	60138	deletion	Uncertain significance	*OR4F5*	Intron-intergenic
chr5:710584-712291	1707	duplication	Benign	*ZDHHC11B*	Exon
chr10:5005570-5005738	168	deletion	Uncertain significance	*AKR1C1*	Intron
chr22:42537580-42539671	2091	duplication	Benign	*CYP2D7*	Exon - Intron
chr22:42899159-42899324	165	duplication	Uncertain significance	*SERHL*	Exon
chr22:42911149-42911319	170	duplication	Benign	*RRP7A*	Exon

Supplementary Table S3. A summary of the CNVs identified only in the ALS twin. It contains information about the size, the type of CNV, the prediction of the effect according to ClassifyCNV, the gene and the position.

Chromosome Coordinates (hg19)	Size	Type	Prediction	Gene	Position
chr1:12595-129201	116606	deletion	Uncertain significance	*DDX11L1-ORF45*	Intergenic
chr1:1643627-1644770	1143	duplication	Benign	*CDK11A*	Exon-Intron
chr1:1653992-1656939	2947	duplication	Uncertain significance	*CDK11A, SLC53E2A*	Intergenic
chr3:197846563-197847695	1130	deletion	Benign	-	Deep intergenic
chr4:337999-338261	262	deletion	Uncertain significance	*ZNF141*	Exon-Intron
chr21:40716983-40717288	305	duplication	Uncertain significance	*HMGN1*	Exon-Intron

Supplementary Table S4. A summary of the CNVs identified only in the healthy co-twin. It contains information about the size, the type of CNV, the prediction of the effect according to ClassifyCNV, the gene and the position.

RNA sequencing results and validation

Supplementary Fig. S1 represents quality controls on produced reads obtained by FastQC.

Supplementary Fig. S2 shows the heatmap of DEGs based on edgeR pvalue adjusted and log2FC values.

A B

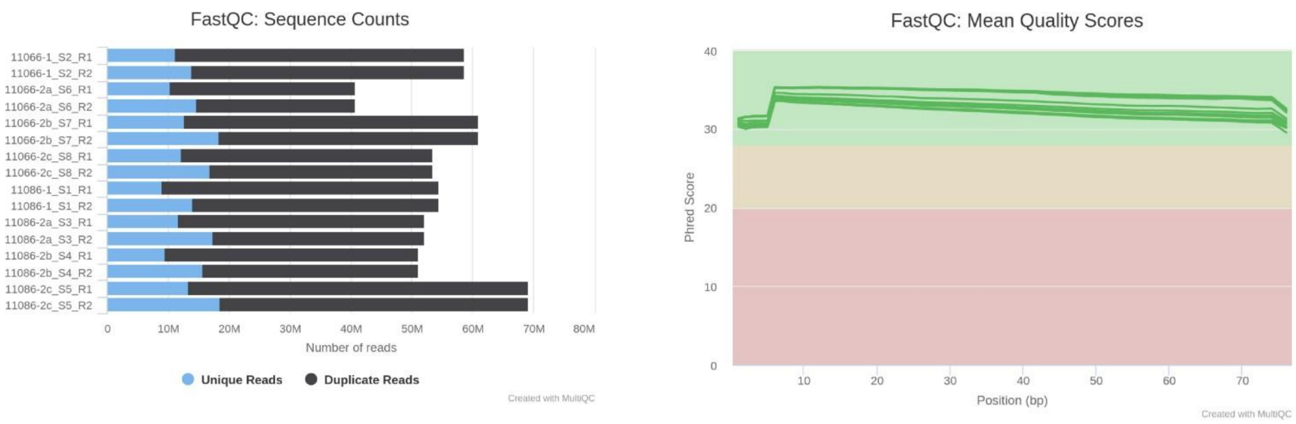

Supplementary Fig. S1: FastQC quality control (Babraham Bioinformatics). (**A**) FastQC sequence counts plot. (**B**) Per base sequence quality plot.

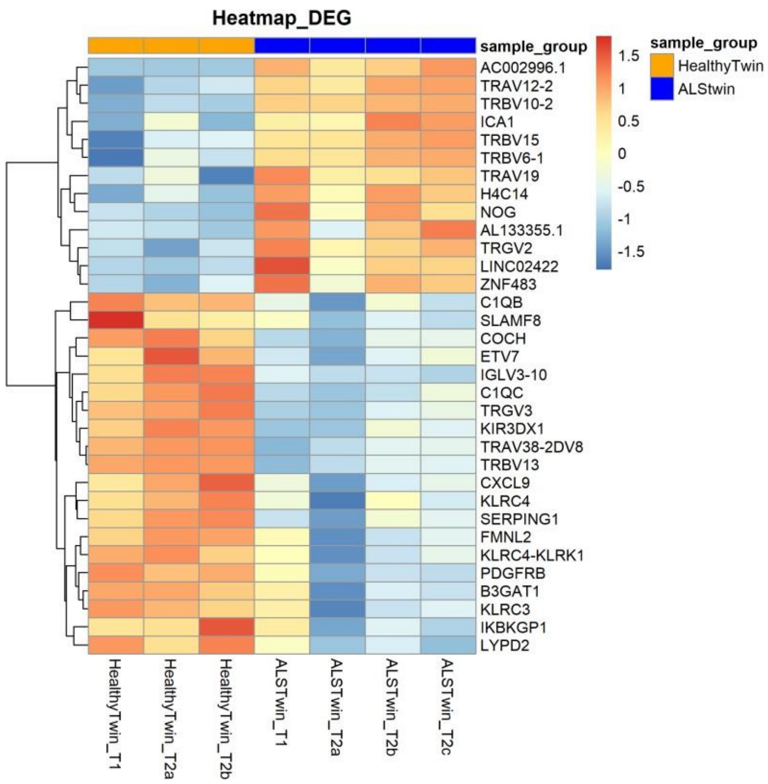

Supplementary Fig. S2. Heatmap showing the identified 33 differentially expressed genes (DEGs) for the healthy twin (left) and the ALS-affected co-twin (right) based on edgeR log2FC and p.adj. Upregulated genes are represented in the red scale, while downregulated genes are depicted in the blue scale. The genes' name is displayed on the right, while clustering of genes by similar expression values is presented on the left.

To validate the transcriptomic results, we performed digital droplet PCR on 3 upregulated (*PF4V1,*

JUN, PKIB) genes obtained by DESeq2 and 3 downregulated (*KLRC3, SERPING, JUN*) genes, obtained by both methods, in the ALS twin compared to the healthy co-twin, based on TPM counts. As observed in the picture below (Supplementary Fig. S3), for *C1QB*, *SERPING1* and *KLRC3* genes the concentration, expressed as copies/µl, in the ALS twin is lower compared to the healthy one. On the other hand, for *JUN, PF4V1* and *PKIB*, the affected twin has a higher concentration than the other twin. Therefore, the upregulation and downregulation of these specific genes detected in the RNAseq analyses were experimentally validated and confirmed using a different semi-quantitative method.

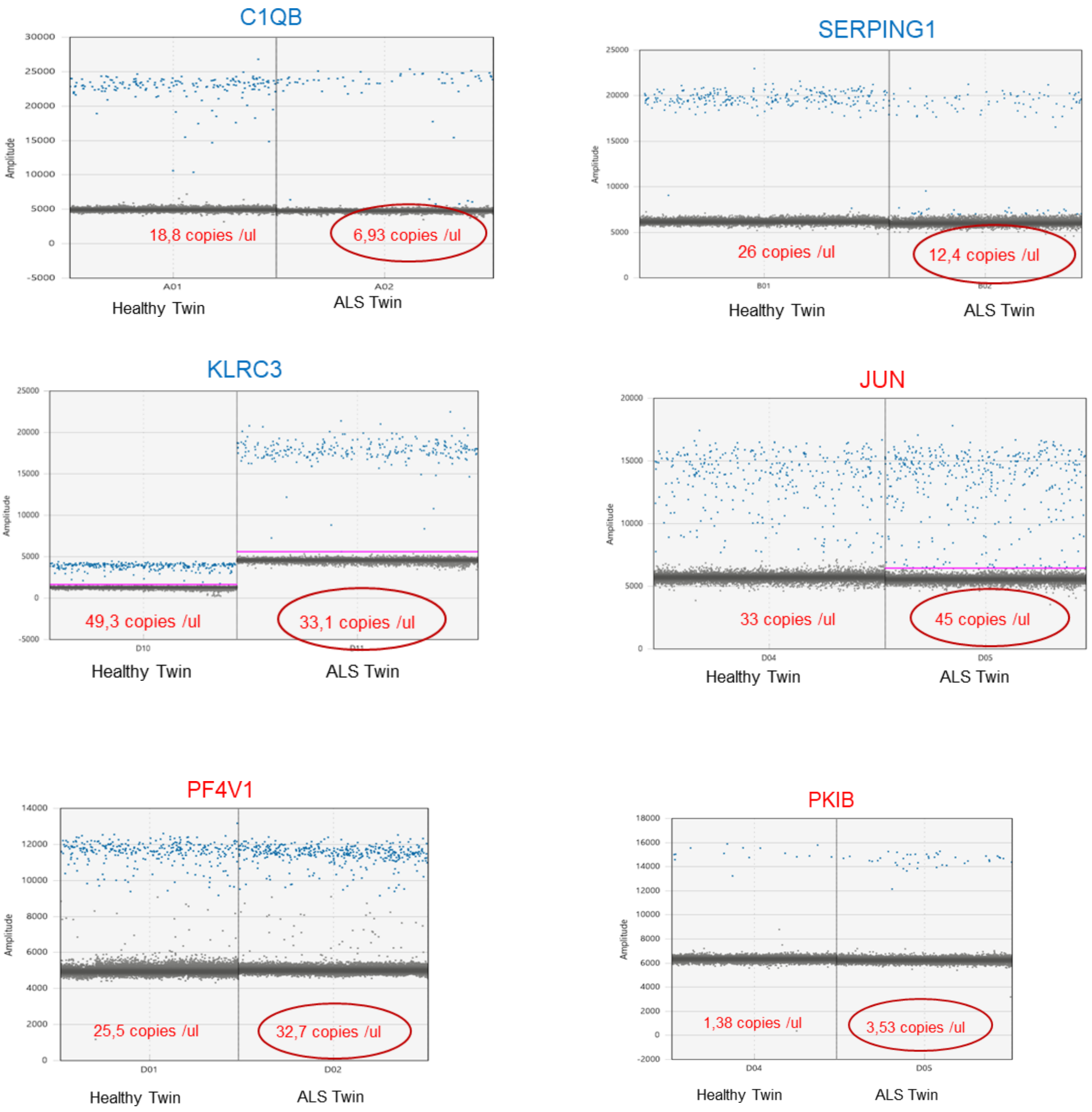

Supplementary Fig. S3. Droplet digital PCR outputs of the 6 genes validated. Blue dots represent the droplets, the X axis corresponds to the samples and Y axis corresponds to the amplitude of the fluorescent signal. The concentration of each gene for the two samples is expressed as copies/ µl. Genes were confirmed to be respectively upregulated or downregulated.

Methylation results and validation

Analysis was performed on Infinium Methylation EPIC Array (Illumina, San Diego, USA). First array quality controls and filters to remove probes that failed with detection with p-value >0.01 or < 3 probes in at least 5% of samples; multi-hit, non-CpG, Chromosome X/Y and SNP-overlapping probes were performed by Illumina GenomeStudio Methylation Module and the Bioconductor package ChAMP (Chip Analysis Methylation Pipeline)[3]. Supplementary Fig. S4 shows the normalized β values distribution plot produced by ChAMP. Normalized β values distribute between 0 and 1, where 0 corresponds to unmethylated signals and 1 to the methylated ones.

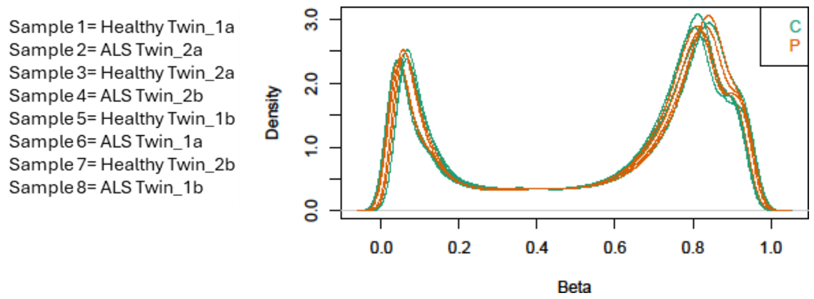

Supplementary Fig. S4. Normalized β values distribution plot produced by ChAMP.

PCA showed the distribution of the eight samples according to their phenotype and position on the array (Group1= position 1-2-3-4; Group2= position 5-6-7-8) (Supplementary Fig.S5 A-B). Since we identified two groups based on sample position on the array (Group 1 and Group 2) with a possible outlier (HealthyTwin_1a) as shown in Supplementary Fig. S5 B, we corrected for batch effect and confirmed HealthyTwin_1a to be an outlier (Supplementary Fig. S5 C-D). After removing the outlier, the analysis was conducted on the remaining seven samples— four samples from the ALS twin and three from the healthy co-twin. After batch correction, we observe that samples primarily distribute according to their disease status (C) rather than technical variables (D), indicating that the batch correction was effective.

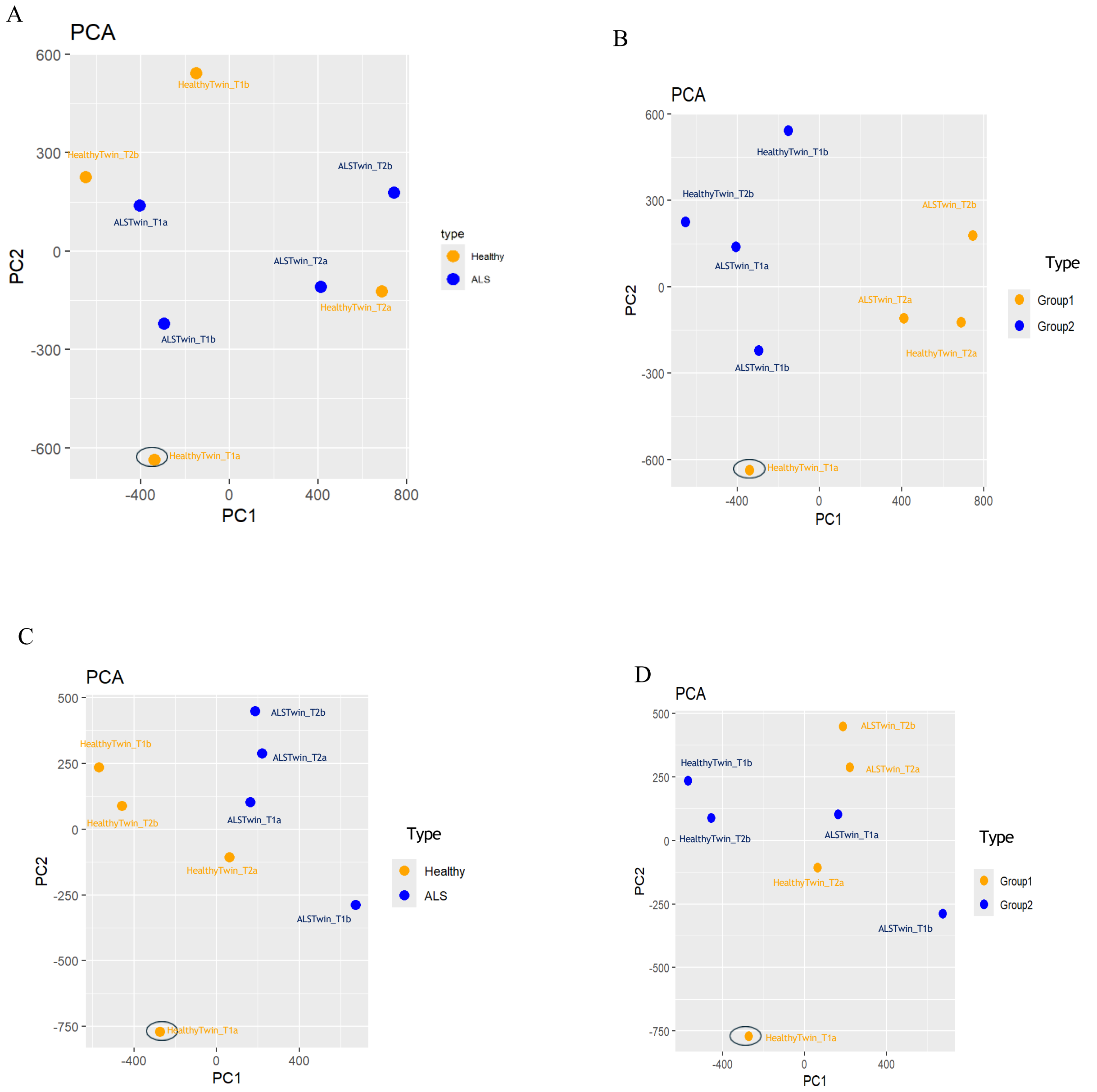

Supplementary Fig. S5. Samples distribution according to Principal Component Analysis (PCA). **(A-B)** The PCAs show the distribution of the eight samples labelled by their disease status and position on the array (Group1= position 1-2-3-4; Group2= position 5-6-7-8). Samples divided into two groups based on the position on the array; **(C-D)** PCAs showing eight samples after correcting for batch effect and labelled by their disease status and position on the array. HealthyTwin_1a was confirmed to be outlier.

By applying filters of adjusted p-value ≤ 0.05 and | Δβ | > 0.06, we identified 250 differentially methylated probes (DMP) mapping in 190 genes: 108 DMPs were hypermethylated and 142 hypomethylated in the ALS twin compared to the healthy co-twin.

The technical validation of epigenetic results was assessed by methylation-specific droplet digital PCR combined with methylation-dependent restriction enzymes (ddMSP). Among all the DMPs identified in the Epic array analysis, we decided to validate cg18454685 that maps in *CACNA1G* and cg27533288 that maps in *VAX* 1. As observed in Supplementary Fig. S6, for both *CACNA1G* and *VAX1* genes the concentration expressed as copies/µl in the ALS twin is lower (48 copies/µl for

CACNA1G and 54 copies/µl for *VAX1*) compared to the concentration found in the healthy one (59 copies/µl for *CACNA1G* and 64 copies/µl for *VAX1*), which confirms the hypomethylation of the two probes and genes in the affected subject.

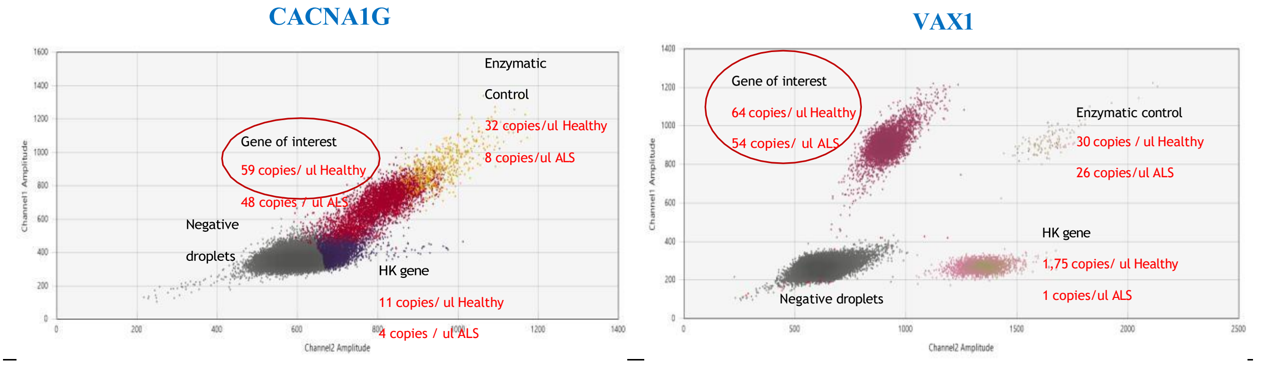

Supplementary Fig. S6. Results of ddMSP. Four separate clouds correspond to the genes considered in the experimental validation: from the top left the gene of interest, the gene to control restriction enzyme activity, housekeeping gene and negative droplets. The concentration of both genes of interest in the affected twin is lower compared to the healthy co-twin, confirming the hypomethylation results observed by EPIC array.

However, these validated signals are listed among the smoking-associated probes. Considering the effect of smoking on DNA methylation and the twins' different smoking status, from the initial betavalue matrix derived from the total eight samples, we removed 63,535 smoking-associated probes[4]. From PCA, we could observe that samples distributed very similarly to the previous condition (Supplementary Fig. S5). Samples were again divided according to their position on the array (Group1= position 1-2-3-4; Group2= position 5-6-7-8) and HealthyTwin_1a was identified as possible outlier (Supplementary Fig. S7 A-B); thus, we corrected for batch effect, confirming it as outlier. Supplementary Fig. S7 C-D show PCA after batch correction: we can observe that samples primarily distribute according to their disease status (C) rather than technical variables (D), indicating that the batch correction was effective.

Then, we removed the outlier and the smoking related probes, and performed a final, new analysis on a total of seven samples. PCA based on normalized data was once again influenced by sample position on the array (Group1= position 2-3-4; Group2= position 5-6-7-8) (Supplementary Fig. S7 E); thus, we corrected for batch effect. Supplementary Fig. S7 F-G show PCA after batch correction: we can observe that samples primarily distribute according to their disease status (F) rather than technical variables (G), indicating that the batch correction was effective.

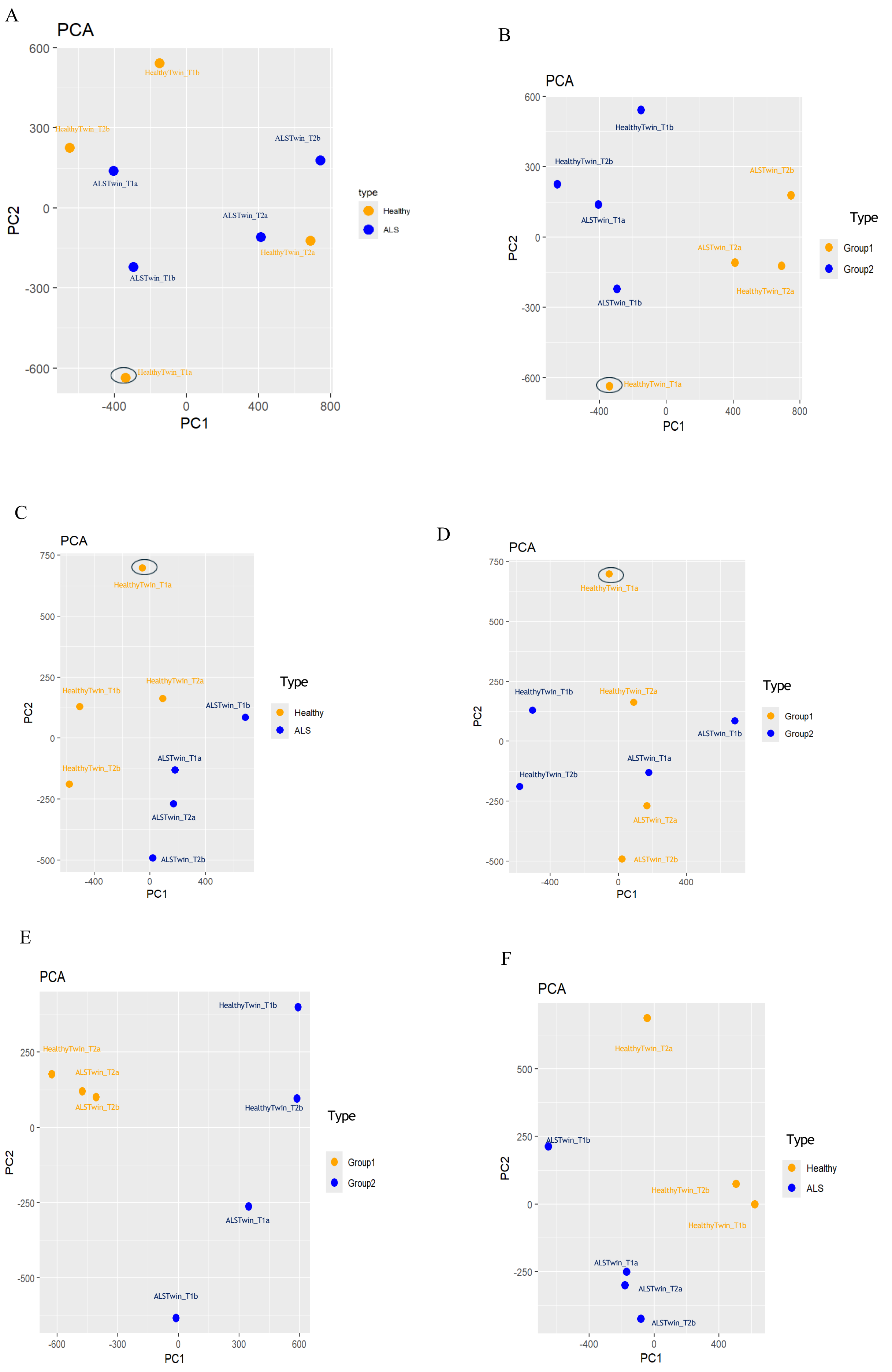
A
PCA
HealthyTwin_T1b
HealthyTwin_T2b
ALSTwin_T2b
ALSTwin_T1a
ALSTwin_T2a
HealthyTwin_T2a
ALSTwin_T1b
HealthyTwin_T1a
600
300
0
-300
-600
-400
0
400
800
PC1
PC2
type
Healthy
ALS
B
PCA
HealthyTwin_T1b
HealthyTwin_T2b
ALSTwin_T2b
ALSTwin_T1a
ALSTwin_T2a
HealthyTwin_T2a
ALSTwin_T1b
HealthyTwin_T1a
600
300
0
-300
-600
-400
0
400
800
PC1
PC2
Type
Group1
Group2
C
PCA
HealthyTwin_T1a
HealthyTwin_T1b
HealthyTwin_T2a
ALSTwin_T1b
ALSTwin_T1a
HealthyTwin_T2b
ALSTwin_T2a
ALSTwin_T2b
750
500
250
0
-250
-500
-400
0
400
PC1
PC2
Type
Healthy
ALS
D
PCA
HealthyTwin_T1a
HealthyTwin_T1b
HealthyTwin_T2a
ALSTwin_T1b
ALSTwin_T1a
HealthyTwin_T2b
ALSTwin_T2a
ALSTwin_T2b
750
500
250
0
-250
-500
-400
0
400
PC1
PC2
Type
Group1
Group2
E
PCA
HealthyTwin_T1b
HealthyTwin_T2a
ALSTwin_T2a
ALSTwin_T2b
HealthyTwin_T2b
ALSTwin_T1a
ALSTwin_T1b
250
0
-250
-500
-600
-300
0
300
600
PC1
PC2
Type
Group1
Group2
F
PCA
HealthyTwin_T2a
ALSTwin_T1b
HealthyTwin_T2b
HealthyTwin_T1b
ALSTwin_T1a
ALSTwin_T2a
ALSTwin_T2b
500
250
0
-250
-400
0
400
PC1
PC2
Type
Healthy
ALS

G

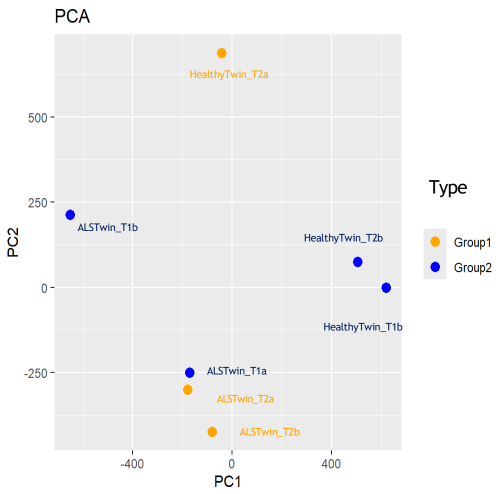

Supplementary Fig. S7. (A-B-C-D): Samples distribution according to Principal Component Analysis (PCA), after removal of the smoking associated probes. **(A-B)** The PCAs show the distribution of the eight samples labelled by their disease status and position on the array (Group1= position 1-2-3-4; Group2= position 5-6-7-8). Samples divided into two groups based on the position on the array; **(C-D)** PCAs showing eight samples after correcting for batch effect and labelled by their disease status and position on the array. HealthyTwin_1a was confirmed to be outlier. **(E-F-G):** Samples distribution according to Principal Component Analysis (PCA), after removal of the smoking associated probes and the outlier. **(E)** 7 samples distribute according to their position on the array (Group1= position 2-3-4; Group2= position 5-6-7-8); **(F-G)** PCA of 7 samples post batch correction: samples are coloured based on the sample group (“Healthy”, “ALS”) and position on the array (Group1= position 2-3-4; Group2= position 5-6-7-8).

By applying filters of adjusted *p*-value ≤ 0.05 and | Δβ | > 0.06 on the remaining 677,794 probes, we identified 89 differentially methylated probes (DMP) mapping in 64 genes (Supplementary File 2). Supplementary Fig. S8 shows two enrichment analyses by Metascape conducted on the 21 hypermethylated and 43 hypomethylated genes.

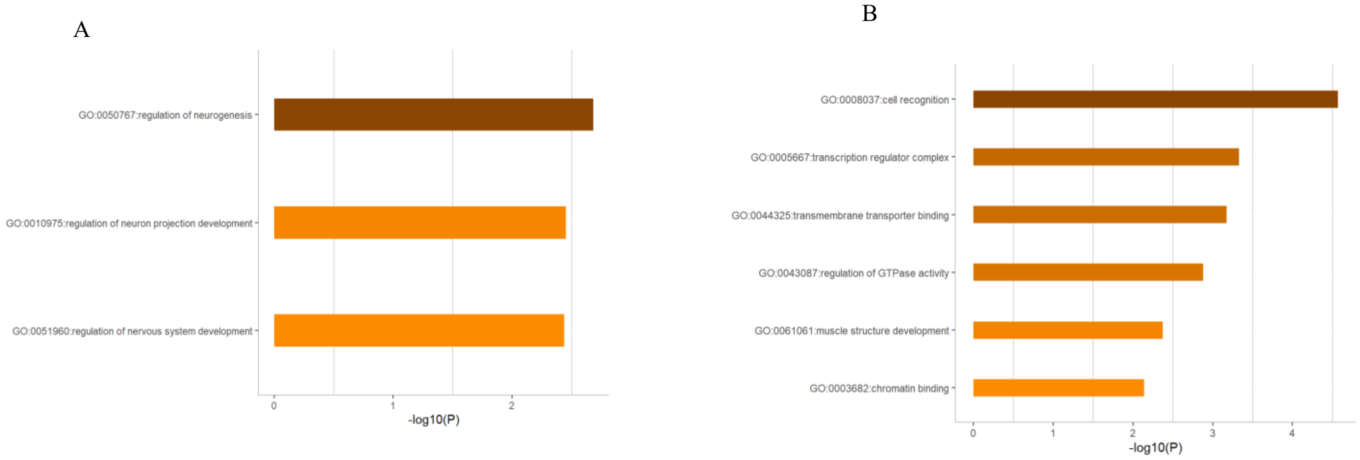

Supplementary Fig. S8. Results of two separate enrichment analyses. **(A)** Result of enrichment analysis on the 21 genes associated to hypermethylated probes. **(B)** Most associated terms derived from enrichment analysis on the 43 genes associated to hypomethylated probes.

Multi omics data integration

After analyzing the three omics individually, we tried to combine genetic, epigenetic and transcriptomic results. Supplementary Fig. S9 shows the Venn diagram resulting from the integration by a late strategy.

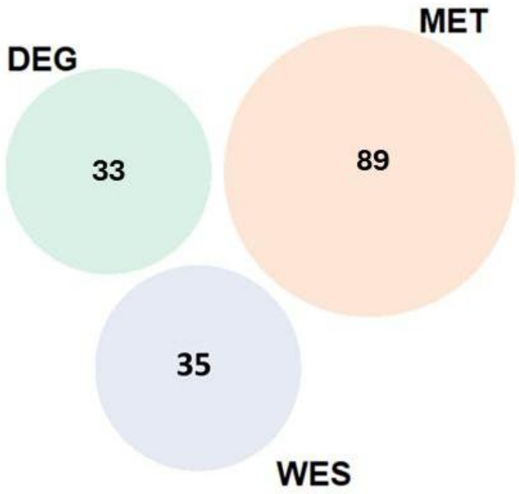

Supplementary Fig. S9. Venn diagram showing the relationship between the three analysed omics: transcriptomics, epigenetics and genetics. There isn't any gene in common between the three datasets.

Data Integration with literature datasets

Reference	Number of Twin Pairs	Available Omics Data
Tarr, I.S., McCann, E.P., Benyamin, B. et al. *Monozygotic twins and triplets discordant for amyotrophic lateral sclerosis display differential methylation and gene expression.* Sci Rep 9, 8254 (2019). https://doi.org/10.1038/s41598-019-44765-4[5]	6 twin pairs	Epigenetic (450K Array) and transcriptomic data; DNAm Age

Young PE, KumJewS, Buckland ME, Pamphlett R,Suter CM(2017) *Epigenetic differences between monozygotic twins discordant for amyotrophic lateral sclerosis (ALS) provide clues to disease pathogenesis*. PLoS ONE12(8): e0182638. https://doi.org/10.1371/journal. pone.0182638[6]	5 twin pairs	Epigenetic data (450K Array and RRBS); DNAm Age
Tazelaar, G.H.P. *et al.* (2023) '*Whole genome sequencing analysis reveals post-zygotic mutation variability in monozygotic twins discordant for amyotrophic lateral sclerosis*', Neurobiology of Aging, 122, pp. 76–87. Available at: https://doi.org/10.1016/j.neurobiolaging.2022.11.010[7]	21 twin pairs	Genetic (WGS) and epigenetic data (450K Array); DNAm Age
Yazar V, Ruf WP, Knehr A, et al. *DNA Methylation Analysis in Monozygotic Twins Discordant for ALS in Blood Cells.* Epigenetics Insights. 2023;16. doi:10.1177/25168657231172159[8]	7 twin pairs	Epigenetic data (850K Array) and DNAm Age
Zhang M, Xi Z, Ghani M, et al. *Genetic and epigenetic study of ALS-discordant identical twins with double mutations in SOD1 and ARHGEF28.* J Neurol Neurosurg Psychiatry 2016;87:1268–1270[9]	1 twin pair	Genetic and epigenetic data (450K Array); DNAm Age
Meltz Steinberg, K., Nicholas, T. J., Koboldt, D. C., Yu, B., Mardis, E., & Pamphlett, R. (2015). *Whole genome analyses reveal no pathogenetic single nucleotide or structural differences between monozygotic twins discordant for amyotrophic lateral sclerosis.* Amyotrophic Lateral Sclerosis and Frontotemporal Degeneration, 16(5–6), 385–392. https://doi.org/10.3109/21678421.2015.1040029[10]	5 twin pairs	Genetic (WGS)

Supplementary Table S5. The table shows the five articles on monozygotic twins or triplets discordant for ALS that we selected in order to compare and possibly confirm the results obtained from our study.

**Reference**	**Number of Twin Pairs**	**Available Omics Data**

Gagliardi, S., Zucca, S., Pandini, C. et al. *Long non-coding and coding RNAs characterization in Peripheral Blood Mononuclear Cells and Spinal Cord from Amyotrophic Lateral Sclerosis patients.* Sci Rep 8, 2378 (2018). https://doi.org/10.1038/s41598-018-20679-5[11]	10 SALS and 3 controls	Transcriptomic data
Feró, O., Varga, D., Nagy, É. et al. *DNA methylome, R-loop and clinical exome profiling of patients with sporadic amyotrophic lateral sclerosis*. *Sci Data* 11, 123 (2024). https://doi.org/10.1038/s41597-024-02985-y[12]	7 SALS and 7 controls	Epigenetic data (RRBS); Genetic data (Clinical Exome Sequencing)
Grima N., Liu S., Southwood D. et al. *RNA sequencing of peripheral blood in amyotrophic lateral sclerosis reveals distinct molecular subtypes: considerations for biomarker discovery.* Neuropathol Appl Neurobiol. 2023; 49(6):e12943. doi:10.1111/nan.12943[13]	96 SALS and 48 controls	Transcriptomic data
Liguori M, Nuzziello N., et al., *Dysregulation of micrornas and target genes networks in peripheral blood of patients with sporadic amyotrophic lateral sclerosis* (2018) Front. Mol. Neurosci. 11:288. doi: 10.3389/fnmol.2018.00288[14]	56 SALS and 20 controls	Transcriptomic data
Garofalo M, Pandini C. et al., *RNA Molecular Signature Profiling in PBMCs of Sporadic ALS Patients: HSP70 Overexpression Is Associated with Nuclear SOD1. Cells.* 2022 Jan 15;11(2):293. doi: 10.3390/cells11020293[15]	18 SALS and 12 controls	Transcriptomic data
Kühlwein JK, Ruf WP, et al., *ALS is imprinted in the chromatin accessibility of blood cells.* Cell Mol Life Sci. 2023 Apr 24;80(5):131. doi: 10.1007/s00018-023-04769-w[16]	23 SALS and 18 controls	Transcriptomic data, ATAC-seq data
Cai Z, Jia X, et al., *Epigenome-wide DNA methylation study of whole blood in patients with sporadic amyotrophic lateral sclerosis*. Chin Med J (Engl). 2022 Jun 20;135(12):1466-1473. doi: 10.1097/CM9.0000000000002090[17]	32 SALS and 32 controls	Epigenetic data
Yang, T., Li, C., et al. *Genome-wide DNA methylation analysis related to ALS patient progression and survival. J Neurol* 271, 2672–2683 (2024). https://doi.org/10.1007/s00415-024-12222-6[18]	41 SALS and 27 controls	Epigenetic data (MC-seq)
Hop PJ, Zwamborn RAJ, Veldink JH, et al., *Genome-wide study of DNA methylation shows alterations in metabolic, inflammatory, and cholesterol pathways in ALS*. Sci Transl Med. 2022 Feb 23;14(633): eabj0264. doi: 10.1126/scitranslmed.abj0264[19]	6763 SALS and 2943 controls	Epigenetic data (450K and EPIC array)

Supplementary Table S6. Articles on sporadic ALS patients that we selected in order to confirm previous findings and identified disease mechanisms.

References

1. Kopanos, C. *et al.* VarSome: the human genomic variant search engine. *Bioinformatics* **35**, 1978–1980 (2019).

2. Gurbich, T. A. & Ilinsky, V. V. ClassifyCNV: a tool for clinical annotation of copy-number variants. *Sci Rep* **10**, 20375 (2020).

3. Tian, Y. *et al.* ChAMP: updated methylation analysis pipeline for Illumina BeadChips. *Bioinformatics* **33**, 3982–3984 (2017).

4. Hoang, T. T. *et al.* Comprehensive evaluation of smoking exposures and their interactions on DNA methylation. *eBioMedicine* **100**, 104956 (2024).

5. Tarr, I. S. *et al.* Monozygotic twins and triplets discordant for amyotrophic lateral sclerosis display differential methylation and gene expression. *Sci Rep* **9**, 8254 (2019).

6. Young, P. E., Kum Jew, S., Buckland, M. E., Pamphlett, R. & Suter, C. M. Epigenetic differences between monozygotic twins discordant for amyotrophic lateral sclerosis (ALS) provide clues to disease pathogenesis. *PLoS ONE* **12**, e0182638 (2017).

7. Tazelaar, G. H. P. *et al.* Whole genome sequencing analysis reveals post-zygotic mutation variability in monozygotic twins discordant for amyotrophic lateral sclerosis. *Neurobiology of Aging* **122**, 76–87 (2023).

8. Yazar, V. *et al.* DNA Methylation Analysis in Monozygotic Twins Discordant for ALS in Blood Cells. *Genet Epigenet* **16**, 251686572311721 (2023).

9. Zhang, M. *et al.* Genetic and epigenetic study of ALS-discordant identical twins with double mutations in *SOD1* and *ARHGEF28*. *J Neurol Neurosurg Psychiatry* **87**, 1268–1270 (2016).

10. Meltz Steinberg, K. *et al.* Whole genome analyses reveal no pathogenetic single nucleotide or structural differences between monozygotic twins discordant for amyotrophic lateral sclerosis. *Amyotrophic Lateral Sclerosis and Frontotemporal Degeneration* **16**, 385–392 (2015).

11. Gagliardi, S. *et al.* Long non-coding and coding RNAs characterization in Peripheral Blood Mononuclear Cells and Spinal Cord from Amyotrophic Lateral Sclerosis patients. *Sci Rep* **8**, 2378 (2018).

12. Feró, O. *et al.* DNA methylome, R-loop and clinical exome profiling of patients with sporadic amyotrophic lateral sclerosis. *Sci Data* **11**, 123 (2024).

13. Grima, N. *et al.* RNA sequencing of peripheral blood in amyotrophic lateral sclerosis reveals distinct molecular subtypes: Considerations for biomarker discovery. *Neuropathology Appl Neurobio* **49**, e12943 (2023).

14. Liguori, M. *et al.* Dysregulation of MicroRNAs and Target Genes Networks in Peripheral Blood of Patients With Sporadic Amyotrophic Lateral Sclerosis. *Frontiers in Molecular Neuroscience* **11**, 288 (2018).

15. Garofalo, M. *et al.* RNA Molecular Signature Profiling in PBMCs of Sporadic ALS Patients: HSP70 Overexpression Is Associated with Nuclear SOD1. *Cells* **11**, 293 (2022).

16. Kühlwein, J. K. *et al.* ALS is imprinted in the chromatin accessibility of blood cells. *Cell. Mol. Life Sci.* **80**, 131 (2023).

17. Cai, Z., Jia, X., Liu, M., Yang, X. & Cui, L. Epigenome-wide DNA methylation study of whole blood in patients with sporadic amyotrophic lateral sclerosis. *Chinese Medical Journal* **135**, 1466–1473 (2022).

18. Yang, T. *et al.* Genome-wide DNA methylation analysis related to ALS patient progression and survival. *J Neurol* **271**, 2672–2683 (2024).

19. Genome-wide study of DNA methylation shows alterations in metabolic, inflammatory, and cholesterol pathways in ALS. https://www.science.org/doi/10.1126/scitranslmed.abj0264 doi:10.1126/scitranslmed.abj0264.
